# Long distance movement of DIR1 and investigation of the role of DIR1-like during systemic acquired resistance in *Arabidopsis*

**DOI:** 10.3389/fpls.2013.00230

**Published:** 2013-07-04

**Authors:** Marc J. Champigny, Marisa Isaacs, Philip Carella, Jennifer Faubert, Pierre R. Fobert, Robin K. Cameron

**Affiliations:** ^1^Department of Biology, McMaster UniversityHamilton, ON, Canada; ^2^Plant Biotechnology InstituteSaskatoon, SK, Canada

**Keywords:** DIR1, systemic acquired resistance, DIR1-like, lipid transfer protein, long distance signaling

## Abstract

DIR1 is a lipid transfer protein (LTP) postulated to complex with and/or chaperone a signal(s) to distant leaves during Systemic Acquired Resistance (SAR) in *Arabidopsis*. DIR1 was detected in phloem sap-enriched petiole exudates collected from wild-type leaves induced for SAR, suggesting that DIR1 gains access to the phloem for movement from the induced leaf. Occasionally the *defective* in *induced resistance1 (dir1-1)* mutant displayed a partially SAR-competent phenotype and a DIR1-sized band in protein gel blots was detected in *dir1-1* exudates suggesting that a highly similar protein, DIR1-like (At5g48490), may contribute to SAR. Recombinant protein studies demonstrated that DIR1 polyclonal antibodies recognize DIR1 and DIR1-like. Homology modeling of DIR1-like using the DIR1-phospholipid crystal structure as template, provides clues as to why the *dir1-1* mutant is rarely SAR-competent. The contribution of DIR1 and DIR1-like during SAR was examined using an *Agrobacterium*-mediated transient expression-SAR assay and an estrogen-inducible DIR1-EGFP/*dir1-1* line. We provide evidence that upon SAR induction, DIR1 moves down the leaf petiole to distant leaves. Our data also suggests that DIR1-like displays a reduced capacity to move to distant leaves during SAR and this may explain why *dir1-1* is occasionally SAR-competent.

## Introduction

Plants respond to pathogen infection locally at the individual cell level and can acquire resistance in tissues distant from the initial site of infection. Acquired resistance in plants was originally documented more than 70 years ago (Chester, 1933) and the term Systemic Acquired Resistance (SAR) was first used by Ross (1961). SAR is defined as a defense response induced by certain local infections resulting in broad-spectrum resistance in distant tissues to normally virulent pathogens (Kuć, 1982).

Research using tobacco, cucumber and *Arabidopsis* demonstrated that the SAR response occurs in stages that include induction, movement of a long distance signal(s), perception of the signal(s) which primes the plant for the manifestation stage in which the plant responds to normally virulent pathogens in a resistant manner [reviewed in Champigny and Cameron (2009)]. Induction of SAR is initiated when a necrotizing pathogen infects a leaf and results in either the formation of a localized hypersensitive response (HR) and local resistance, or in disease-induced necrosis (Kuć, 1982). However, recent studies in tobacco (Liu et al., 2010a) and *Arabidopsis* (Mishina and Zeier, 2007) suggest that cell death is not required to induce SAR.

Grafting experiments with cucumber provided evidence that a long distance signal moves from induced rootstocks to distant scions (Jenns and Kuć, 1979). Moreover, girdling with hot cotton wool in cucumber (Guedes et al., 1980) or by removing the stem sheath in tobacco (Tuzun and Kuć, 1985) prevented signal transport to distant leaves, suggesting that the SAR long distance signal(s) moves via the phloem. However, these techniques reduce both phloem and cell-to-cell movement, indicating that the SAR long distance signal could travel using either or both transportation routes. Source-sink relationships (orthostichies) in the *Arabidopsis* rosette were investigated in relation to SAR-competence (Kiefer and Slusarenko, 2003). Movement of the SAR signal from induced to distant leaves to establish and manifest SAR as measured by *PR-1* expression and reduced growth of *Pseudomonas syringae* pv. *tomato* (*Pst)*, indicated that upper leaves in and outside the orthostichy of the lower induced leaf were also SAR-competent. These data suggest that the *Arabidopsis* long distance SAR signal(s) moves via the phloem and other means, perhaps cell-to-cell.

The discovery that salicylic acid (SA) levels rise in phloem exudates of induced tobacco (Malamy et al., 1990) and cucumber (Métraux et al., 1990) led to the hypothesis that SA may be a SAR long distance signal (Uknes et al., 1992). Cucumber leaf detachment experiments (Rasmussen et al., 1991) as well as grafting studies with transgenic tobacco that accumulates little SA strongly suggested that SA is not a SAR long distance signal, but is required in distant tissue during the priming and manifestation stages of the SAR pathway (Gaffney et al., 1993; Vernooij et al., 1994; Pallas et al., 1996).

The establishment phase of SAR involves the perception of the mobile signal(s) in distant tissue, resulting in a primed state that is correlated with the accumulation of inactive protein kinases and chromatin modifications in SAR-associated gene promoters and is thought to provide the molecular memory of priming [reviewed in Conrath (2011)]. Manifestation of SAR is associated with the expression and activity of a set of *PR* genes (van Loon and van Strien, 1999). The rapid and abundant accumulation of these defense proteins during the manifestation stage may be the molecular basis for systemic resistance [reviewed in Champigny and Cameron (2009), Shah and Zeier (2013)].

A number of genes acting at the initiation or terminal stages of the SAR pathway have been identified [reviewed in Durrant and Dong (2004), Vlot et al. (2008)]. Key among these is *NPR1*, whose function is required for the SA-dependent expression of PR proteins [reviewed in Durrant and Dong (2004)]. Recent work suggests that NPR1 is a receptor for SA (Wu et al., 2012) and that the paralogous proteins NPR3 and NPR4 may also act as SA receptors in *Arabidopsis* leaves during the manifestation stage of SAR (Fu et al., 2012). Information about long distance signaling during SAR was obtained from the study of *dir1-1* (*defective* in *induced resistance1*). Petiole exudates, enriched for phloem sap and/or molecules that move cell-to-cell down the petiole, collected from induced, but not mock-inoculated wild-type leaves were effective in eliciting expression of *PR-1* when infiltrated into wild-type or *dir1-1* plants, indicating that long-distance SAR signals are present in wild-type exudates and *dir1-1* can perceive these signals. Exudates similarly collected from *dir1-1* leaves did not induce *PR-1* expression in wild-type leaves, suggesting that *dir1-1* is defective either in the synthesis of the SAR mobile signal or its long-distance transport to distant leaves (Maldonado et al., 2002). These data and the fact that *DIR1* encodes a putative lipid transfer protein (LTP) led to the hypothesis that DIR1 is involved in long distance signaling and may chaperone a lipid signal to distant leaves during SAR (Maldonado et al., 2002).

LTPs are ubiquitous in plants and are associated with many developmental and stress response processes (Yeats and Rose, 2008). The structures of a number of LTPs have been determined, revealing that they possess a consensus motif of eight cysteine residues engaged in four disulphide bridges forming a central hydrophobic cavity which can bind long fatty acid chains (Yeats and Rose, 2008). Lascombe et al. (2006, 2008) determined the structure and lipid binding properties of DIR1 expressed in the yeast *Pichia pastoris* using fluorescence and X-ray diffraction. DIR1 shares some structural and lipid binding properties with the LTP2 family but unique to DIR1 is its ability to bind two monoacylated phospholipids *in vitro*. Studies of glycerolipid biosynthesis mutants (Nandi et al., 2004; Chaturvedi et al., 2008) also suggest that a lipid-derived molecule is a long distance SAR signal. Other studies indicate that methyl salicylate (MeSA) azelaic acid (AA), glycerol-3-phospate (G3P)-derived factor, and dehydroabietinal (DA) may also be SAR long distance signals (Park et al., 2007; Vlot et al., 2008; Jung et al., 2009; Chanda et al., 2011; Chaturvedi et al., 2012). The SAR-promoting role of these small molecules requires the presence of DIR1 protein as demonstrated by the inability of G3P, AA, MeSA, or DA to induce SAR in *dir1-1*, suggesting that one or more of these molecules may be physiological ligands of DIR1. Overexpression/SAR studies in *dir1-1* demonstrated that two tobacco DIR1 orthologs are functionally redundant to Arabidopsis DIR1 and thus DIR1 is important for SAR in both *Arabidopsis* and tobacco (Liu et al., 2011a). Furthermore, a putative tomato DIR1 ortholog was identified in untreated tomato phloem by protein gel blot analysis, however its importance in the tomato SAR response has yet to be established (Mitton et al., 2009).

Expression and localization of DIR1 using DIR1pro:GUS and DIR1 pro:DIR1-GUS fusion lines (Champigny et al., 2011) demonstrated that DIR1 is expressed in seedlings and flowers, and ubiquitously in untreated or mock-inoculated mature leaf cells including phloem sieve elements and companion cells. Intercellular washing fluid (IWF) experiments and subcellular localization of transiently expressed DIR1:EGFP in tobacco indicated that DIR1's ER signal sequence targets it for secretion to the cell wall. Interestingly, a transgenic line expressing DIR1 without its signal sequence in which DIR1 accumulates in the cytosol rescued the *dir1-1* SAR defect, suggesting that a cytosolic pool of DIR1 is important for SAR (Champigny et al., 2011).

Previously we hypothesized that DIR1 moves to distant tissues during SAR (Maldonado et al., 2002) and recently demonstrated that DIR1 is well situated to participate in long distance signaling as it is expressed in companion cells and sieve elements (Champigny et al., 2011). Numerous reviews (Durrant and Dong, 2004; Parker, 2009; Dempsey and Klessig, 2012) present models in which DIR1 translocates to distant tissues during SAR. Chanda et al. (2011) provide evidence for the movement of ectopically expressed *Arabidopsis* DIR1-EGFP in *N. benthamiana* plants in response to G3P infiltration. However, the movement of native DIR1 during biologically induced SAR has not been demonstrated. Therefore, we conducted experiments to determine if DIR1 possesses the key characteristic of a SAR long distance signal: the ability to move from induced to distant leaves during the SAR response. We also sought to distinguish the role of DIR1 and the highly similar DIR1-like protein by developing and using a transient *Agrobacterium*-SAR assay and an estrogen-inducible DIR1-EGFP/*dir1-1* line.

## Results

### DIR1 is detected in petiole exudates collected from leaves induced for SAR

**Figure 1:**
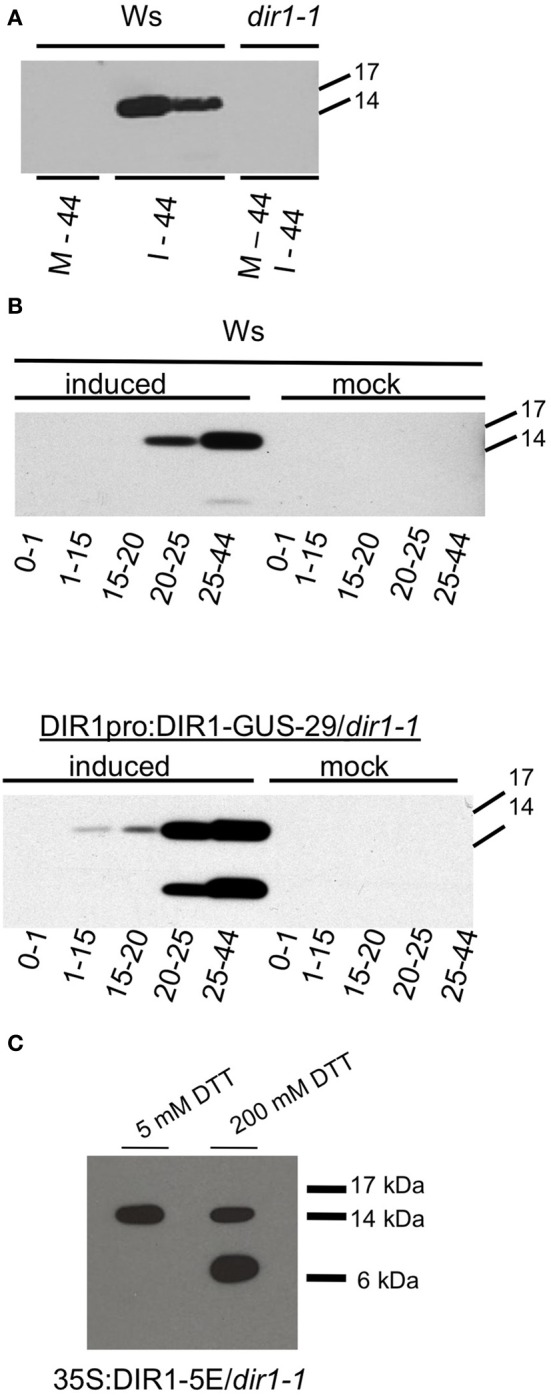
**Detection of DIR1 in petiole exudates. (A)** Ws and *dir1-1* plants were mock-inoculated or inoculated with SAR-inducing *Pst (avrRpt2)* (10^6^ cfu ml^−1^). Seven petioles per tube were allowed to exude starting at ~8 hpi until 44 hpi (= one exudate). Each exudate was lyophilized and subjected to protein gel blot analysis with the DIR1 antibody. M-44 = mock-inoculated petioles exuded for 44 hpi, I-44 = petiole from leaves induced for SAR exuded for 44 hpi. **(B)** Ws and DIR1pro:DIR1-GUS-29/*dir1-1* were mock-inoculated or induced for SAR with *Pst (avrRpt2)* (10^6^ cfu ml^−1^), petiole exudates were collected from ~8 hpi for 0–1 h, then transferred to another tube for 1–15 h, transferred again for 15–20, then for 20–25 and 25–44 hpi (denoted by arrows). Exudates were lyophilized and subjected to SDS-PAGE. **(C)** Exudates collected over 44 hpi with SAR-inducing *Pst (avrRpt2)* (10^6^ cfu ml^−1^) from the DIR1 overexpression line (35S:DIR1-5E/*dir1-1*) were lyophyilized and resuspended in 5 or 200 mM dithiothreitol (DTT) and subjected to protein gel blot analysis with the DIR1 antibody. Protein molecular weight markers are indicated (17,14, 6 kDA). **(A,B)** have been repeated with similar results three times. **(C)** has been repeated once with similar results.

Using petiole exudate infiltration experiments, Maldonado et al. (2002) demonstrated that SAR-inducing signals are present in wild-type, but not *dir1-1* plants, leading to the hypothesis that DIR1, a putative LTP, binds a lipid or hydrophobic molecule and participates in the long distance signaling stage of SAR. If DIR1 chaperones a hydrophobic signal(s) or is part of a signal complex [DIR1 plus hydrophobic molecule(s)] that translocates to distant tissue during SAR, then it should be possible to detect DIR1 in petiole exudates collected throughout the SAR induction stage. Mock-inoculated leaves and leaves induced for SAR with *Pst (avrRpt2)* were collected from wild-type (Ws-2) and *dir1-1* mutant plants at 10 h post inoculation (hpi), quickly surface sterilized and immersed in 1 mM EDTA to prevent sieve element blockage at the cut petiole ends (King and Zeevaart, 1974). Petioles were allowed to exude over 44 h. Petiole exudate protein levels were determined, followed by concentration by lyophilization and protein gel blot analysis with an anti-DIR1 polyclonal antibody (Maldonado et al., 2002). Exudates collected from mock-inoculated leaves consistently contained less protein per exudate (~3 μg ml^−1^) compared to exudates collected from leaves induced for SAR (~30 μg ml^−1^), suggesting that additional proteins enter the phloem during SAR induction (Figure S1). A DIR1 antibody signal of approximately 15 kDa was detected in Ws exudates collected from leaves induced for SAR, but not in mock-inoculated Ws or *dir1-1* exudates (Figure 1A). The absence of DIR1 antibody signals in exudates collected from mock-inoculated leaves is consistent with experiments done with untreated Arabidopsis petiole exudates performed by Guelette et al. (2012). A DIR1 signal is not present at detectable levels in exudates prior to the induction of SAR suggesting that DIR1 protein moves out of the leaf blade and down the petiole during the SAR induction stage. Later experiments (Figures 5, 6) indicate that *Agrobacterium tumefaciens* does not induce SAR and does not elicit the accumulation of DIR1 antibody signals (Agro-inoculated, followed by mock-inoculated treatments). Therefore, the DIR1 antibody signals observed in petiole exudates collected from SAR-induced leaves are specific to the SAR response.

If DIR1 protein is moving down the petiole during SAR induction, collection of exudates at different times after induction should provide information about the length of time it takes DIR1 to move. Exudates from mock-inoculated leaves and leaves induced for SAR were collected from Ws and a DIR1-GUS transgenic line (DIR1pro:DIR1-GUS-29/*dir1-1*, Champigny et al., 2011) to follow DIR1 movement using protein gel blot analysis and assaying for GUS activity of the DIR1-GUS fusion protein. At 8 hpi, mock-inoculated and SAR-induced leaves were collected and allowed to exude for 1 h, transferred to new tubes and allowed to exude from 1 to 15 h, transferred again and allowed to exude for 15–20 h, then 20–25 and finally 25–44 h, followed by protein gel blot analysis (Figure 1B). The 0–1 h exudates were collected to demonstrate that the DIR1 signal observed was not due to proteins leaking from wounded and dying cells before the wound response sealed the non-phloem cells at the cut petiole ends. A DIR1 signal of ~15 kDa was not observed in any of the mock-inoculated samples or in the 0–1 h exudates collected from leaves induced for SAR. DIR1 protein was observed in SAR-induced exudates at 20–25 hpi in Ws and at 1–15 and 15–20 hpi in the DIR1-GUS line. Bands of ~7 and 15 kDa were observed in exudates from the DIR1-GUS line (Figure 1B). Mature DIR1 is comprised of 77 amino acids with a predicted molecular weight of ~7 kDa (Lascombe et al., 2008). The presence of 7 and ~15 kDa bands suggests that DIR1 is present in petiole exudates in both monomeric and dimeric forms. Typically, the 15 kDa form was observed in IWFs (Figure S2). During SDS-PAGE, samples are heated in SDS to disrupt non-covalent bonds leading to protein denaturation. A reducing agent such as dithiothreitol (DTT) is added to reduce covalent disulfide bonds, however some disulfide bonds are not broken at the DTT concentrations normally used in denaturing SDS-PAGE gels (5 mM DTT) (Mahler et al., 2009). Therefore, exudates collected from an over-expression line (35S:DIR1/*dir1-1*) were denatured in freshly prepared sample buffer containing either 5 or 200 mM DTT before protein gel blot analysis. A DIR1 band of ~15 kDa was observed in 5 mM DTT and both 7 and 15 kDa bands were observed when the exudate was incubated in 200 mM DTT (Figure 1C), suggesting that the 15 kDa signal represents a DIR1-containing dimer held together by disulfide bonds.

A DIR1-GUS fusion protein (7 or 15 + 68 kDa = 75 or 83) was not detected in petiole exudates (Figure 1B) or in IWFs collected from the DIR1-GUS line (Figure S1). In a few experiments, a ~75 kDa DIR1-GUS band was detected in whole leaf extracts from DIR1-GUS lines using anti-DIR1 and anti-GUS antibodies (Figure S3). Observing low levels of DIR1-GUS in leaf extracts is consistent with our previous results in which a DIR1 signal was not detected in wild-type Ws leaf extracts (Maldonado et al., 2002). These observations, and the fact that GUS activity was detected intracellularly in leaf cells in both the DIR1pro:GUS as well as the DIR1-GUS fusion lines (Champigny et al., 2011) suggests that GUS is cleaved from DIR1 after secretion to the cell wall and/or at some point during SAR induction.

### A DIR1 signal is sometimes detected in *dir1-1* petiole exudates from SAR-induced leaves

**Figure 2:**
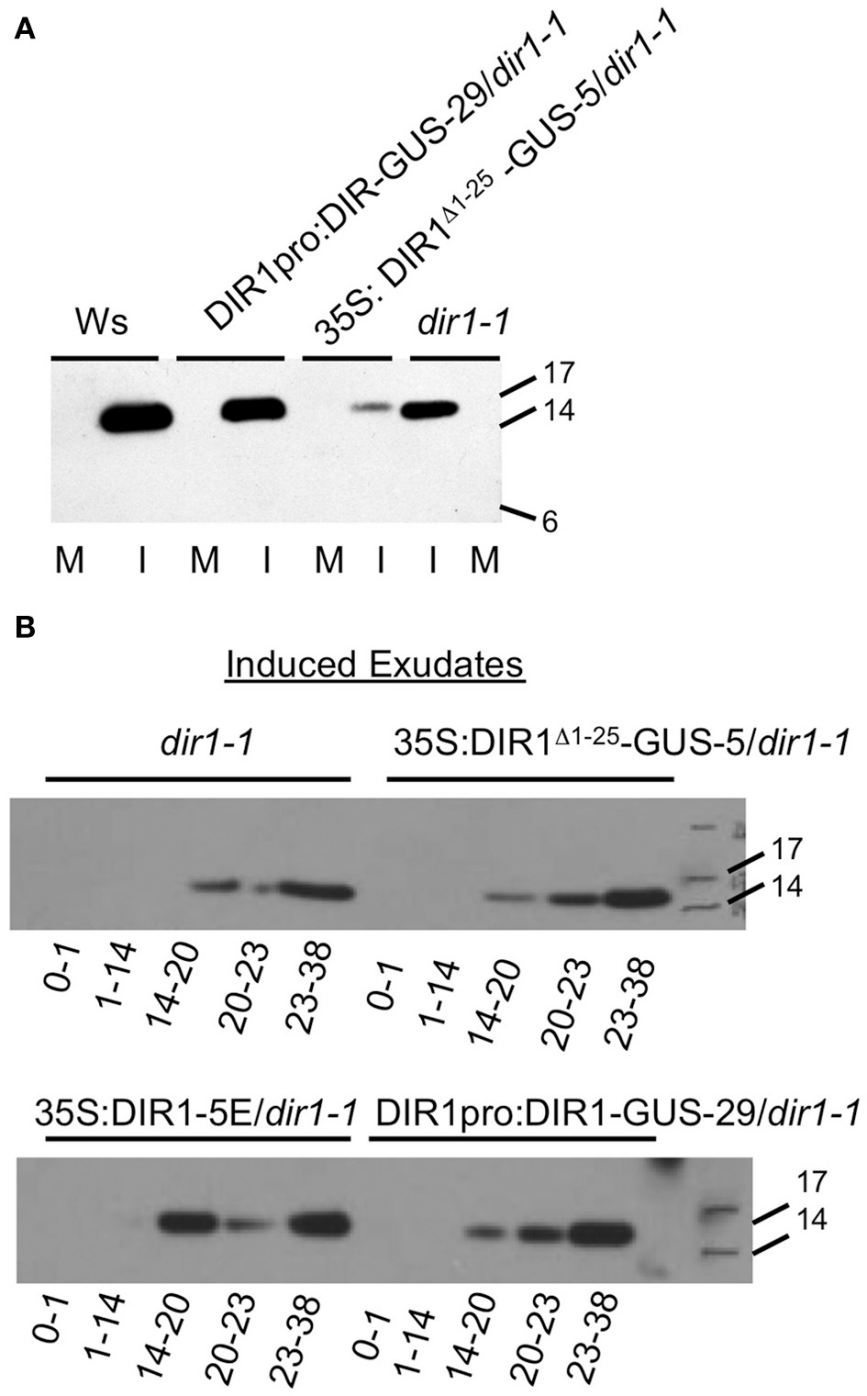
**Detection of a DIR1 antibody signal in petiole exudates collected from *dir1-1* plants.** Petiole exudates were collected from mock-inoculated (M) and SAR-induced (I) (10^6^ cfu ml^−1^ *Pst (avrRpt2*) leaves of DIR1pro:DIR1-GUS-29/*dir1-1*, 35S: DIR1^Δ 1-25^-GUS-5/*dir1-1*, Ws and *dir1-1*, then lyophilized and subjected protein gel blot analysis with the DIR1 antibody. **(A)** Petioles exuded from 8 to 44 hpi **(B)**. SAR-induced leaves were collected at various times following inoculation and allowed to exude for the indicated amount of time (0–1, 1–14, 14–20, 20–23, 23–38 hpi). Protein molecular weight markers are indicated (17, 14, 6 kDA), rLTP—recombinant LTP made in *E. coli*). These experiments were repeated twice with similar results.

In some exudate-protein gel blot experiments, a DIR1-sized band was observed in exudates collected from *dir1-1* leaves induced for SAR. This was observed in experiments similar to Figure 1A in which petioles exuded from 8 to 44 h, or in time course experiments similar to those shown in Figure 1B. A representative experiment is displayed in Figure 2A in which a DIR1-antibody signal was detected in exudates collected from a number of SAR-induced plants including *dir1-1*. Genotyping ruled out seed contamination or loss of the T-DNA insertion in the *dir1-1* gene during self-fertilization over a number of generations, indicating that the DIR1 antibody signal in *dir1-1* exudates is not due to a wild-type *DIR1* allele (data not shown). We also considered that the DIR1 signal observed in *dir1-1* could be the result of a cross-reactive protein accumulating in exudates due to EDTA-induced tissue softening causing cell leakage along the length of submerged petioles (Hepler, 2005). To avoid this issue, we modified the petiole exudate collection method by shortening the exudation time to reduce leakage from petiole cells during exudation.

As shown in Figure 2B, exudate collection began and ended at various times after inoculation (0–1 h, 1–14, 14–20, 20–23, 23–38) of *dir1-1* and the *dir1-1* transgenic lines (35S:DIR1^Δ 1−25^-GUS-5, DIR1pro:DIR1-GUS-29, 35S:DIR1-5E), followed by protein gel blot analysis. Mock-inoculated exudates contained no DIR1 signal (data not shown), while a DIR1 band of ~15 kDa was observed in the 14–20, 20–23, and 23–38 hpi exudates in *dir1-1* and the transgenic lines (35S:DIR1^Δ 1−25^-GUS-5, DIR1pro:DIR1-GUS-29, 35S:DIR1-5E). The timing of the appearance of the protein gel blot signal was similar in the transgenics and in *dir1-1* and no signal was detected in the early exudates (0–1, 1–14 hpi). Even though the same pattern was observed in *dir1-1* and transgenic exudates, *dir1-1* was SAR-defective in this experiment, while the transgenic lines were SAR-competent (SAR assays reported in Figure 6 in Champigny et al., 2011). Because we observed a DIR1 antibody signal in *dir1-1* using short and long exudation methods, we hypothesize that the 15 kDa DIR1-antibody signal in SAR-induced *dir1-1* results from the movement of a DIR1-like protein down the petiole where it exudes from sieve elements at the petiole ends.

**Figure 3:**
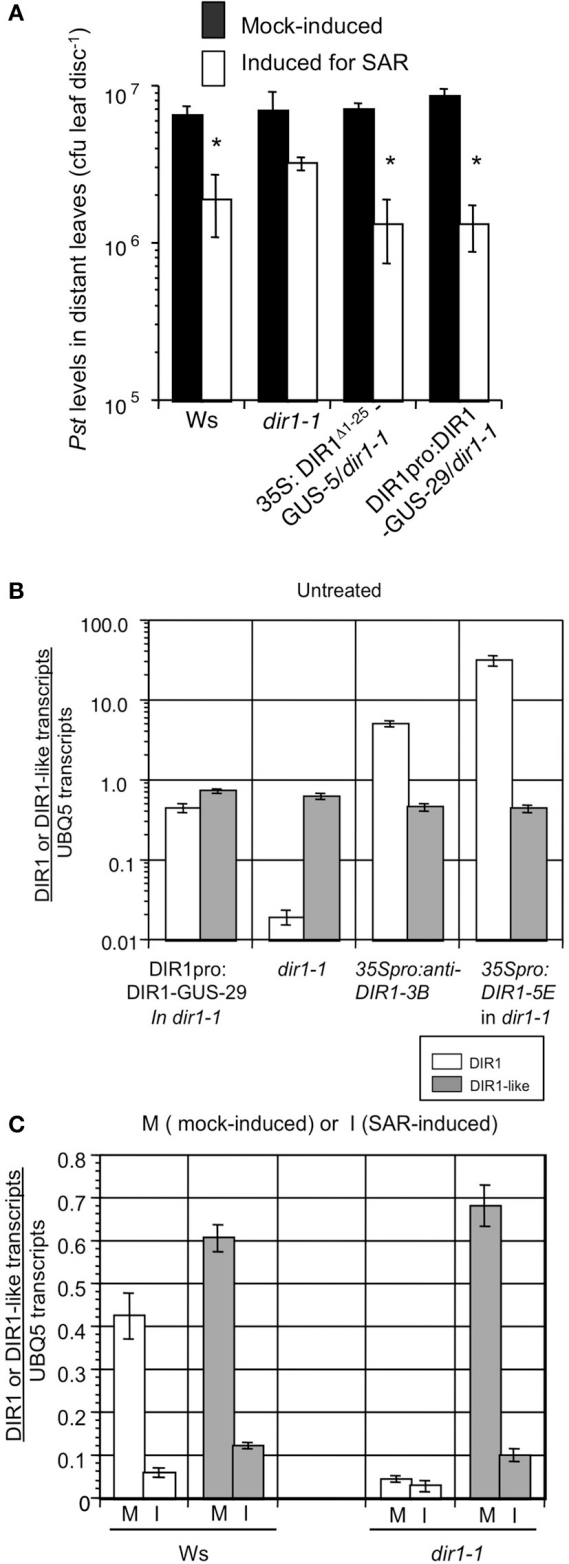
**A partial SAR response is sometimes observed in *dir1-1.* (A)** SAR assays were conducted on Ws, *dir1-1*, DIR1pro:DIR1-GUS-29/*dir1-1* and 35S: DIR1^Δ 1-25^-GUS-5/*dir1-1* by inoculating with 10 mM MgCl_2_ (mock-induced) or inducing for SAR with *Pst (avrRpt2)* (SAR-induced) in 1–2 lower leaves, followed by challenge inoculation with virulent *Pst* in distant leaves 2 days later. Bacterial levels were determined in challenged leaves 3 dpi. Asterisks (^*^) denote a significant difference (student's *t*-test, *p* < 0.05) in bacterial levels between challenged distant leaves of mock- and SAR-induced plants. These experiments were repeated numerous times and a partial SAR response was occasionally observed in *dir1-1* (see text for details). Expression of *DIR1* and *DIR1-like* genes. Real-time RT-PCR analysis was performed using 3 week-old *Arabidopsis* leaves. Expression of *DIR1* is represented by white bars and expression of *DIR1-like* is represented by gray bars. Absolute quantification of transcripts followed the method of Rutledge and Stewart (2008). Error bars represent the standard deviation of four measurements. **(B)** Expression of *DIR1* and *DIR1-like* genes in untreated leaves collected from DIR1pro:DIR1-GUS-29*/dir1-1*, *dir1-1*, 35S:antisenseDIR1-3B/Ws and 35S:DIR1-5E/*dir1-1*. Note the logarithmic scale and PCR primers used do not distinguish between wild-type DIR1 transcript and the antisense version. **(C)** Expression of *DIR1* and *DIR1-like* genes in mock-inoculated (M) and SAR-induced (I) leaves (10^6^ cfu ml^−1^ *Pst-avrRpt2*) harvested at 10 hpi. Experiments were performed twice using real-time RT-PCR.

In support of this hypothesis, *dir1-1* was modestly or partially SAR-competent in some experiments, such that plants induced with *Pst (avrRpt2)* supported modestly lower bacterial levels compared to mock-inoculated *dir1-1*; an example is presented in Figure 3A. Of 30 SAR assays performed with *dir1-1* in our former lab at the University of Toronto over 7 years (′96–′02), *dir1-1* was modestly SAR-competent in two of 30 statistically significant experiments (student's *t*-test) or 6.7% of the time. Of 16 SAR assays performed at McMaster over 4 years (′03–′06), *dir1-1* was modestly SAR-competent in 3 of 16 statistically significant experiments (*t*-test) or 18.8% of the time. These data collected over many years in two different labs led us to speculate that a DIR1-like protein encoded in the *Arabidopsis* genome could be responsible for the DIR1-sized band observed in *dir1-1* exudates and this DIR1-like protein may sometimes compensate for the SAR defect in *dir1-1*.

### Identification of a DIR1-like gene

In our experiments at the University of Toronto and McMaster, no event or environmental factor could be identified as the cause of the modestly SAR-competent phenotype in *dir1-1* observed in some experiments. Therefore, we hypothesized that environmental conditions such as different growth chambers and/or water supply at McMaster University may have been responsible for observing a modest SAR response in *dir1-1* more frequently compared to our lab at U. of Toronto. One explanation that could account for these observations is that a DIR1-like LTP may be present in the *Arabidopsis* genome and partially compensate for the *dir1-1* SAR defect in some circumstances. Another possible explanation is that the T-DNA insertion in the 3′ untranslated region (UTR) of *dir1-1* (Maldonado et al., 2002) does not completely abolish *DIR1* expression such that a small amount of DIR1 is made in *dir1-1* and this may be sufficient to elicit a partial SAR response on some occasions. When DIR1 was first identified (Maldonado et al., 2002), a highly similar LTP was not annotated in the *Arabidopsis* genome (TIGR 2 & 3). A blast search of later genome releases revealed a highly similar gene to *DIR1*, At5g48490. Currently TAIR 10 indicates that *Arabidopsis* encodes at least 70 putative LTPs including DIR1 and At5g48490. The most statistically relevant BLAST hit using *DIR1* (At5g48485) as the query sequence is At5g48490, the adjacent locus on chromosome 5. We refer to this gene as *DIR1-like*. Alignment of the coding and protein sequences indicated that these genes are highly conserved, exhibiting 71% sequence identity and 85% similarity at the amino acid level (Figure S4). Much of the observed variation is the result of differences in the poorly conserved ER signal peptides that are not present in the mature proteins. Analysis of the mature protein sequence revealed an 81% amino acid identity and 88% amino acid similarity.

### DIR1 phylogeny

DIR1-like and DIR1 likely arose through a tandem gene duplication as suggested by Boutrot et al. (2008) and their 88% amino acid sequence similarity (EMBOSS Needleman-Wunsch pairwise alignment EMBL-EBI) and tandem location on chromosome 5. A BLAST search of the *Arabidopsis* genome did not reveal other highly similar genes and a Needleman-Wunsch pairwise global alignment using EMBL-EBI confirmed that all other LTP2s share less than 52% sequence similarity compared to DIR1. Using various *Brassicaceae* family members, a phylogeny of putative DIR1 orthologs was constructed to add support to the hypothesis of tandem duplication as well as to determine the evolutionary node where DIR1 duplication occurred (Figures S5, S13). The *Arabidopsis LTP2* gene (At5g38170) was used as an outgroup based on its low sequence similarity to *DIR1* (37%). Using phylogenetic analysis, two distinct groups are revealed, those with two DIR1 orthologs (*Arabidopsis thaliana* and *lyrata*) and those with one (*Thellungiella salsuginea* and *Brassica rapa*). Because only a single “DIR1-type” gene is present in *T. salsuginea* and *B. rapa*, a tandem duplication event occurring in the last common ancestor of *A. thaliana* and *A. lyrata* likely resulted in the DIR1 and DIR1-like paralogs. Recent species phylogenies of the Brassicaceae family (Schranz et al., 2007) and the DIR1 phylogeny presented here share a similar pattern providing further support for the DIR1 phylogeny. These results are consistent with Boutrot et al. (2008) and provide further information on the timing of DIR1 and DIR1-like duplication in the mustard family.

### Expression of DIR1 and DIR1-like genes

If DIR1-like can occasionally compensate for the absence of DIR1 in the *dir1-1* mutant, then *DIR1-like* should be expressed in *dir1-1*. *DIR1* and *DIR1-like* expression was analyzed in plants of various genotypes at 10 h post-mock-inoculation or SAR induction with *Pst (avrRpt2*), or in untreated leaves. RNA was extracted from leaves of 3-week old plants, reverse transcribed to cDNA and subjected to real-time kinetic RT-PCR (Rutledge and Stewart, 2008) using primer pairs specific for *DIR1* and *DIR1-like*.

Similar to previous studies (Maldonado et al., 2002; Champigny et al., 2011), modest expression of *DIR1* in Ws was further reduced ~10-fold at 10 h post SAR-induction compared to mock-inoculated leaves (from 0.4 to 0.04 *DIR1* transcripts per *UBQ5*), while few *DIR1* transcripts (<0.05 per *UBQ5*) were detected in untreated, mock- or SAR-induced *dir1-1* leaves (Figures 3B,C). As expected, *DIR1* expression was elevated in untreated leaves of the 35S promoter transgenic lines (35S:DIR1 in *dir1-1*, 35S:anti-senseDIR1-3B in Ws) compared to the DIR1 promoter line (DIR1pro:DIR1-GUS in *dir1-1)*. *DIR1-like* transcript levels were similar in all genotypes (0.6–0.8 transcripts per *UBQ5*, Figure 3B) indicating that expression of *DIR1-like* was not influenced by gene silencing effects from the *DIR1* transgenes or by the T-DNA insertion in *dir1-1* near the *DIR1-like* locus. Similar to *DIR1*, *DIR1-like* was modestly expressed in Ws and its expression was further reduced ~6-fold in leaves induced for SAR compared to mock-inoculated Ws and *dir1-1* leaves (Figures 3C, S10B). This suggests that *DIR1-like* expression is suppressed by effectors secreted by *Pst* as was observed for DIR1 (Champigny et al., 2011). *DIR1* and *DIR1-like* show similar expression profiles in untreated, mock or SAR-induced Ws plants and *DIR1-like* expression is unaffected in the *dir1-1* mutant. Therefore, DIR1-like is present in *dir1-1* at DIR1 wild-type levels and could be responsible for the modest SAR response observed in *dir1-1* in some experiments.

### Homology modeling of DIR1-like using the DIR1 crystal structure

**Figure 4:**
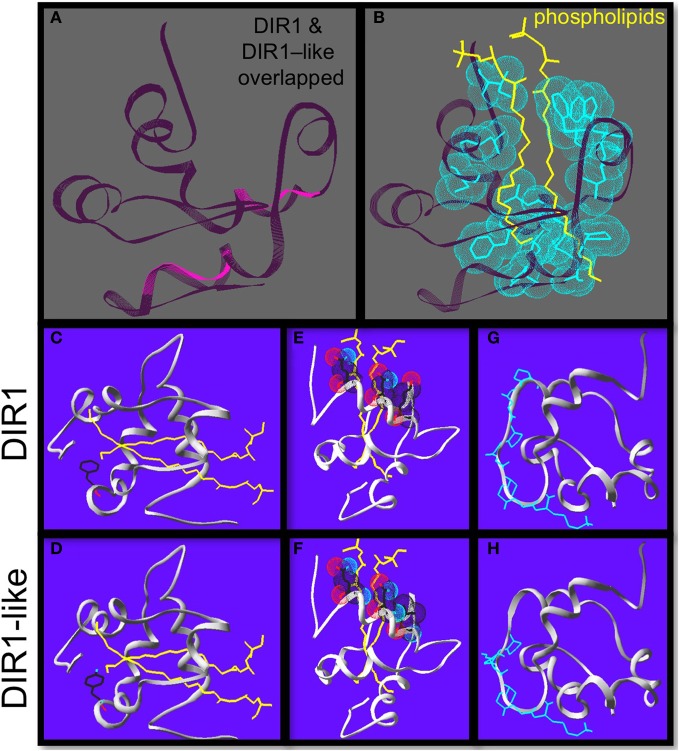
**Homology modeling reveals differences between DIR1 and DIR1-like protein structure.** Homology modeling of DIR1-like protein using the DIR1 crystal structure as a template using the Swiss-pdb viewer 4.0.1 to compare the DIR1 structure and the DIR1-like protein model. **(A)** DIR1 protein backbone in dark purple is over-laid on the DIR1-like protein backbone in pink. **(B)** The two yellow phospholipids extend into the internal lipid binding pocket of DIR1. The 13 hydrophobic residues that make up the lipid binding pocket are highlighted with light blue van der waals forces. **(C–F)** Phospholipids in yellow, oxygen in light blue, nitrogen in red. DIR1 has a hydrophobic non-polar phenylalanine residue **(C)** while DIR1-like has a polar tyrosine residue at the same position **(D)**. at the bottom of the internal lipid binding pocket. **(E,D)** The amino acids at the cavity entrance are shown with their van der waals forces. Three polar residues of DIR1 **(D)** compared to two of DIR1-like **(E)** cup the polar phosphate groups of the phospholipids. The putative SH3 binding motifs of DIR1 and DIR1-like are illustrated in light blue **(G,H)**.

A homology model of DIR1-like was produced by SWISS-MODEL server (Peitsch, 1995; Schwede et al., 2003; Arnold et al., 2006; Kiefer et al., 2009) using the DIR1-phospholipid crystal structure (Lascombe et al., 2006, 2008) as a template to obtain clues to support the idea that DIR1-like sometimes compensates for the SAR defect in *dir1-1*. The Swiss-pdb viewer 4.0.1 (Guex and Peitsch, 1997) was used to compare the DIR1 structure and the DIR1-like protein model. The backbones of both proteins (lacking their ER signal sequences) were overlapped to observe vicinity information on conserved vs. non-conserved residues. Both proteins are very similar in terms of the arrangement of the five α-helices and four disulphide bonds that produce the internal cavity of DIR1 (Figure 4A). A number of interesting differences were observed between DIR1 and the DIR1-like model and amino acid positions are based on the mature protein sequence lacking the signal peptide. Within the binding pocket, thirteen hydrophobic residues were within 3.8 Å of the two phospholipids found in the internal cavity of the DIR1 crystal structure (Figure 4B). A phenylalanine is present at residue 40 in the internal cavity of DIR1, whereas a tyrosine residue was observed in DIR1-like (Figures 4C,D). The polar hydroxyl group present on DIR1-like's tyrosine may reduce the interaction with the phospholipid acyl chains at the bottom of the internal cavity or change the shape of the cavity by pulling toward the polar solution. Additionally, DIR1 has three polar amino acids (GLN9, ASN13, LYS16) located at the entrance of the internal cavity, while DIR1-like has only two (GLN9, ASN13) (Figures 4E,F). Lascombe et al. (2008) postulate that these three polar amino acids create a favorable environment for the hydrophilic phospholipid head groups. Loss of lysine at the cavity entrance in DIR1-like may affect its ability to form a stable interaction with a signal molecule(s) and reduce its capacity to contribute to SAR. Finally, DIR1-like possesses a putative SH3 interaction domain, PXXP, while DIR1 contains PXXPXXP at the same location on the protein surface (Figures 4G,H). SH3 interaction domains act as protein docking sites for transient protein-protein interactions and repeated PXXP motifs strengthen these interactions (Williamson, 1994). Therefore, it is possible that DIR1-like interacts less strongly with a binding partner and this may reduce its ability to contribute to SAR.

### The polyclonal DIR1 antibody recognizes DIR1-like

Homology modeling of DIR1 and DIR1-like suggest that DIR1-like is structurally similar to DIR1. The extensive amino acid sequence similarity between the two proteins raised the possibility that the DIR1 polyclonal antibody recognizes similar epitopes in both proteins. To test this idea, DIR1, DIR1-like and a representative *Arabidopsis* member of the LTP2 family (AT5G38170) were expressed as S-tagged proteins, extracted from *Escherichia coli* cells and subjected to protein gel blot analysis using DIR1 and S-tag antibodies. *Arabidopsis* LTP2 was chosen because its corresponding ortholog in wheat has been crystallized and has a general LTP2 structure similar to DIR1 in terms of the arrangement of α helices and four disulfide bridges (Hoh et al., 2005). Using the S-tag antibody, all three proteins were detected at the expected molecular weight of ~13 kDa. Both DIR1 and DIR1-like were recognized by the DIR1 antibody (Figure S6) while LTP2 (AT5G38170) was not. These data indicate that the DIR1 antibody does recognize DIR1-like, but does not recognize an LTP in the same family.

### Agrobacterium-mediated transient expression/SAR assay

Our petiole exudate data (Figures 1, 2) suggests that DIR1 moves to distant leaves via the phloem, however, DIR1 is expressed constitutively in all living leaf and petiole cells (Champigny et al., 2011), making it difficult to distinguish between endogenous constitutive DIR1 expression and SAR-induced DIR1 movement. Therefore, an *Agrobacterium tumefaciens*-mediated transient expression/SAR assay (Agro-SAR) was developed to overcome this issue. By expressing DIR1 in just one *dir1-1* leaf, followed by SAR induction of the same leaf, we can monitor DIR1 movement to distant leaves in the *dir1-1* mutant which expresses negligible DIR1. *Agrobacterium* T-DNA constructs were created that encode EYFP or a DIR1-EYFP fusion protein containing the ER signal sequence under the control of the 35S promoter. It takes approximately 4 days for *Agrobacterium* to transfer and transiently express its T-DNA containing the gene of interest in infected *Arabidopsis* cells (Wroblewski et al., 2005). Using RT-PCR, we observed low levels of *DIR1-EYFP* expression in *Agrobacterium*-inoculated leaves (Figure S7) consistent with other reports (Tsuda et al., 2012).

**Figure 5:**
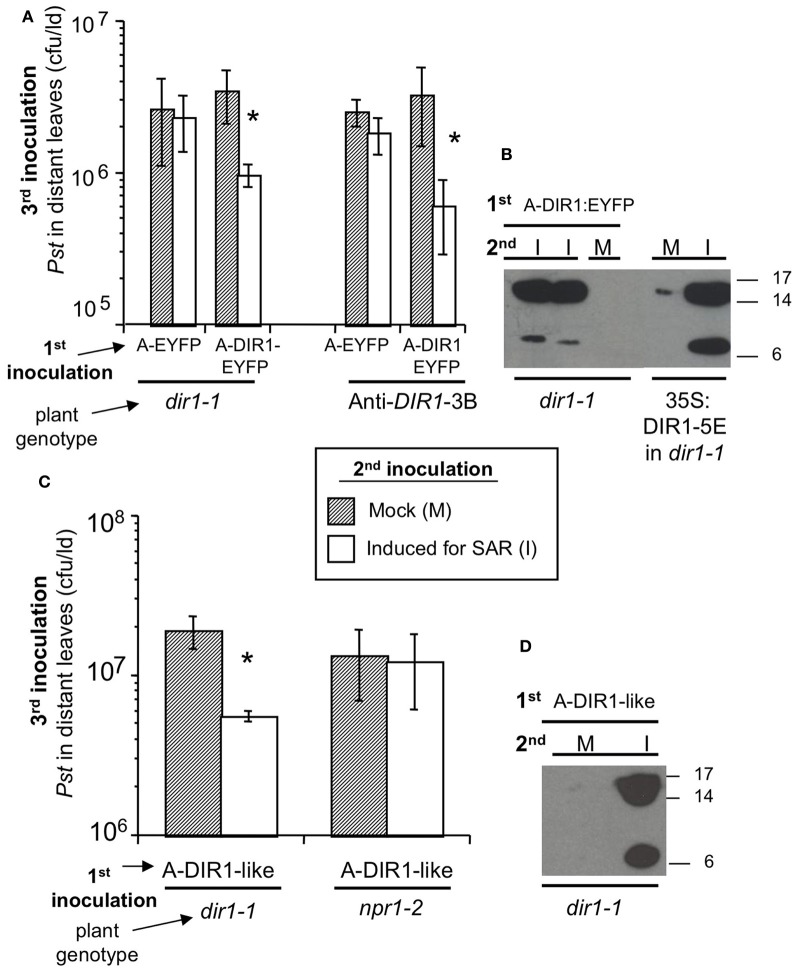
**Transient expression of DIR1 and DIR1-like in one leaf rescues the SAR defect in *dir1-1*. (A)** *dir1-1* and 35S:antisense*DIR1*-3B (Anti-DIR1-3B) plants were subjected to the Agro-SAR assay. 1st inoculation in 2 leaves with either *Agrobacterium* (Agro) containing EYFP or DIR1-EYFP, then a 2nd inoculation 4 days later in the same leaves with either 10 mM MgCl_2_ (Mock) or 10^6^ cfu ml^−1^ *Pst (avrRpt2)* (Induced for SAR). Two days later, one set of plants received a 3rd inoculation with virulent *Pst* (10^5^ cfu ml^−1^) in distant leaves and *Pst* levels were measured 3 dpi. Asterisks (^*^) denote a significant difference (student's *t*-test) in *Pst* levels between mock-inoculated and SAR-induced plants. **(B)** Petiole exudates were collected (20–44 hpi) from another set of plants that were inoculated 1st with Agro DIR1-EYFP and 2nd with either 10 mM MgCl_2_ (M- mock) or *Pst (avrRpt2)* (I—induced for SAR). These exudates were lyophilized and subjected to protein gel blot analysis with the DIR1 antibody. Exudates from SAR-induced 35S:DIR1-5E/*dir1-1* were used as a positive control. **(C)** *dir1-1* and *npr1-2* were subjected to the Agro-SAR assay as in **(A)** using Agro containing DIR1-like. **(D)** Petiole exudates were collected from *dir1-1* plants that were inoculated 1st with Agro DIR1-like, then a 2nd inoculation with mock (M) or SAR-induced (I). Protein molecular weight markers are indicated (17, 14, 6 kDa). **(A–D)** were repeated two additional times with similar results.

Before investigating DIR1-EYFP movement, we assessed the functionality of the Agro-SAR assay by testing the ability of transiently expressed DIR1-EYFP to rescue the SAR-defect in *dir1-1*. The Agro-SAR assay (illustrated in Figure S8) was performed as follows. Two leaves per plant were inoculated with the appropriate *Agrobacterium* strain, followed 4 days later by inoculation of the same leaf with SAR-inducing *Pst (avrRpt2)* or 10 mM MgCl_2_ (mock-inoculation). Two days later, distant leaves were inoculated with virulent *Pst*, followed by determination of *Pst* levels in the distant leaves 3 days later. *Agrobacterium* encoding *35S:EYFP* or *35S:DIR1-EYFP* was inoculated into *dir1-1* or another *dir1* mutant line (*35S:antisenseDIR1-3B*, Maldonado et al., 2002) followed 4 days later with the SAR assay. The SAR defect in both *dir1-1* and the anti-sense *DIR1* line was not rescued when EYFP was expressed, as demonstrated by high *Pst* levels in distant leaves (Figure 5A). Therefore, inoculation with *Agrobacterium* and/or expression of EYFP *in planta* did not rescue the SAR response in either DIR1-deficient genotype. DIR1-EYFP expression via *Agrobacterium*-mediated transient transformation rescued the SAR defect as demonstrated by a 3.5-fold or 5-fold reduction in *Pst* levels in SAR-induced vs. mock-inoculated *dir1-1* or 35S:anti-sense*DIR1*-3B, respectively (Figure 5A). Our results indicate that *Agrobacterium*-mediated transient gene expression can be combined with the SAR assay. Additionally, expression of DIR1 in one leaf followed by SAR-induction is sufficient to rescue the SAR defect in *dir1* mutants.

During this experiment, petiole exudates were collected (Figure 5A) with the aim of monitoring the movement of fluorescent DIR1-EYFP in the *dir1-1* background. Exudates were collected from leaves expressing empty T-DNA vector, EYFP or DIR1-EYFP that were either mock-inoculated or induced for SAR. Fluorescence levels were similar in all exudate samples examined, including exudates collected from untreated leaves (data not shown), suggesting that endogenous fluorescent plant compounds were being detected rather than EYFP fluorescence. RT-PCR data indicated that *DIR1-EYFP* was being expressed in leaves (Figure S7) suggesting that either insufficient DIR1-EYFP was made or EYFP was being cleaved from DIR1 *in planta*. To address this question, Agro-SAR assay exudates were subjected to protein gel blot analysis with DIR1 antibody to determine if the DIR1-EYFP fusion protein was present. Petiole exudates collected from *dir1-1* leaves transiently expressing DIR1-EYFP that were also SAR-induced, contained a ~7 and ~15 kDa DIR1 signal, while mock-inoculated exudates contained no DIR1 signal (Figure 5B). A DIR1-EYFP fusion band (7 or 15 + 26 kDa EYFP) of 33 or 41 kDa was not detected suggesting that EYFP was cleaved from the DIR1 protein. Although it was not possible to track DIR1 using the EYFP tag, this assay provided data that corroborates the idea that DIR1 moves to distant tissues during SAR. However, it was not possible to distinguish DIR1 from DIR1-like as the DIR1-EYFP fusion was not detected in the protein gel blot analysis.

### Agrobacterium-mediated transient expression of DIR1-like rescues the *dir1-1* SAR defect

DIR1-like's involvement in SAR was investigated by comparing DIR1-EYFP and DIR1-like in *dir1-1* Agro-SAR rescue assays. Since DIR1-GUS and DIR1-EYFP fusions could not be detected in SAR-induced petiole exudates (Figures 2, 5), we chose to ectopically express DIR1-like without a reporter. Additionally, the *npr1-2* SAR mutant was included in this assay as a negative control for the SAR response and also to determine if NPR1 acts downstream of DIR1 in the SAR pathway. *Agrobacterium* encoding *DIR1-like* was inoculated into *dir1-1* or *npr1-2* plants followed by the SAR assay 4 days later. *Pst* levels were reduced 4-fold in SAR-induced compared to mock-inoculated *dir1-1* transiently expressing native (signal sequence-containing) DIR1-like (Figure 5C). Thus, ectopic expression of DIR1-like in one leaf of the *dir1-1* mutant compensated for the *dir1-1* SAR defect. However, expression of DIR1-like (Figure 5C) or DIR1-EYFP (Figure 7A) in *npr1-2* did not rescue the *npr1-2* SAR-defect suggesting that DIR1 and DIR1-like act upstream of NPR1 in the SAR pathway. Interestingly, SAR-induced exudates collected from *dir1-1* plants expressing DIR1-like displayed a DIR1-antibody signal in protein gel blot experiments (Figure 5D), suggesting that similar to DIR1, DIR1-like moves down the petiole during SAR induction.

### DIR1-antibody signals are detected in petiole exudates of distant tissues using the agro-SAR assay

**Figure 6:**
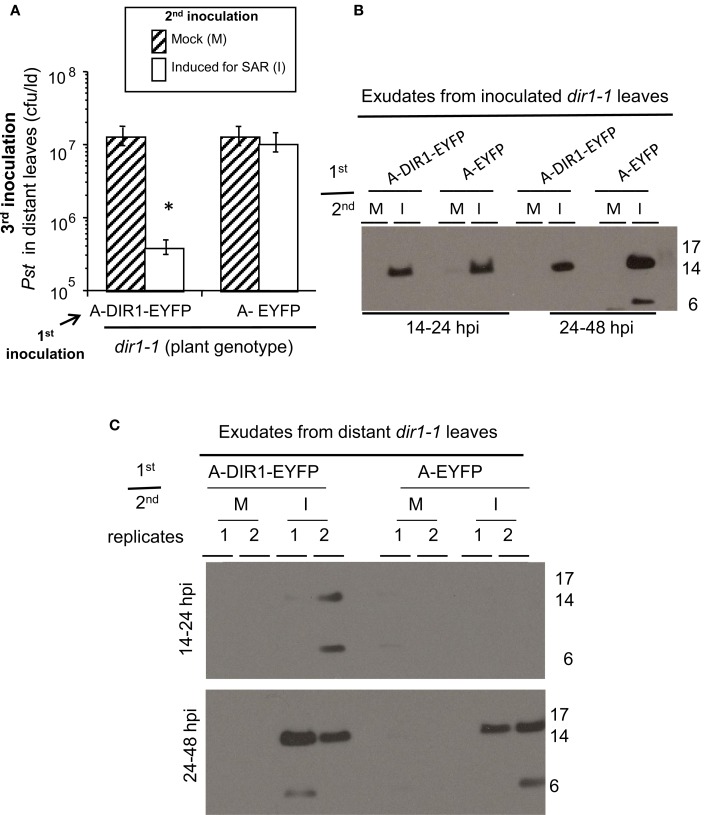
**DIR1-EYFP Agro-SAR assay and DIR1-antibody signals in distant leaf exudates. (A)** The Agro-SAR assay was performed as illustrated in Figure S8. Petiole exudates were collected (14–48 hpi) from lower leaves (Inoc) which received a 1st inoculation with either Agro EYFP or DIR1-EYFP, followed by a 2nd inoculation that was either mock (M) or with SAR-inducing *Pst-avrRpt2*. Exudates were also collected from distant leaves (Dis) of these same plants. Exudates from inoculated **(B)** or distant leaves **(C)** were lyophilized and subjected to protein gel blot analysis with DIR1 antibody. This experiment was repeated twice with similar results. ^*^denotes a significant difference (Student's *t*-test, *p* < 0.05).

**Figure 7:**
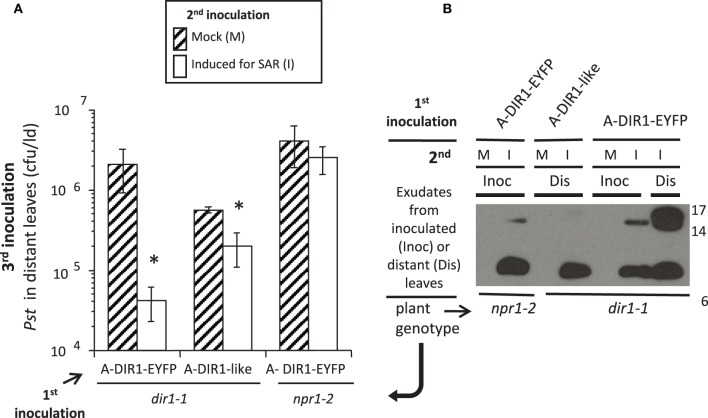
**DIR1-like Agro-SAR assay and DIR1-antibody signals in distant leaf petiole exudates. (A)** The Agro-SAR assay was performed as in Figure S8. **(B)** Petiole exudates were collected (20–46 hpi) from mock-inoculated (M) or SAR-induced (I) lower leaves (Inoc) and from distant leaves (Dis). Exudates were lyophilized and subjected to protein gel blot analysis with DIR1 antibody. Exudates from mock-inoculated distant leaves expressing DIR1-EYFP were analyzed separately (DIR1 antibody signal not observed). Protein molecular weight markers are indicated (17, 14, 6 kDa). This experiment was repeated twice with similar results. ^*^denotes a significant difference (Student's *t*-test, *p* < 0.05).

If DIR1 and DIR1-like are long distance signals during SAR, then these proteins should move not only down the petioles of induced leaves, but from these petioles to distant leaves to initiate the establishment/priming stage of SAR. This hypothesis was tested by performing Agro-SAR assays with *35S:DIR1-EYFP* or *35S:DIR1-like* and collecting exudates starting at 24 hpi until 46 hpi from distant *npr1-2* or *dir1-1* leaves. The amount of DIR1 in whole leaf extracts collected from induced (Maldonado et al., 2002) and distant leaves (Figure S12) was undetectable by protein gel blot analysis. Therefore, distant leaf exudates were collected and concentrated to observe DIR1 and DIR1-like movement to distant tissues. Expression of DIR1-EYFP in *dir1-1* followed by SAR induction elicited a robust (>10-fold) SAR response compared to mock-inoculated plants (Figure 7A). A less robust, but statistically significant (student's *t*-test) 4-fold reduction in *Pst* levels was observed in SAR-induced compared to mock-inoculated plants expressing DIR1-like, and as expected, expression of DIR1-EYFP did not rescue the SAR defect in *npr1-2* (Figure 7A). Exudates collected from leaves that were first inoculated with either *Agrobacterium* containing *35S:DIR1-EYFP*, *35S:DIR1-like* or *35S:EYFP* followed by mock-inoculation, contained no DIR1-antibody signal (Figures 6B, 7B). DIR1-antibody signals appeared to be more abundant in *dir1-1* expressing DIR1-EYFP compared to DIR1*-like* in both induced and distant leaf petiole exudates (Figure 7B). In experiments comparing DIR1-EYFP and EYFP transient expression, DIR1 antibody signals in induced leaf exudates were similar (Figures 6B, S9), whereas DIR1-sized bands were observed only at the later timepoint (24–48 hpi) in distant leaf exudates of plants expressing EYFP (Figure 6C). Since the DIR1 antibody recognizes DIR1-like (Figure S6) and DIR1-like is expressed in *dir1-1* (Figure 3), we reason that the DIR1 antibody signal in *dir1-1* plants transiently expressing EYFP is due to endogenous DIR1-like. Taken together, the induced and distant leaf exudate data supports the idea that the occasional SAR-competent phenotype observed in *dir1-1* could be due to DIR1-like's reduced capacity to move to distant leaves.

### Movement of DIR1-EGFP is observed during SAR using estrogen inducible transgenic DIR1-EGFP lines

Our protein gel blot data using DIR1-GUS transgenics or *Agrobacterium*-mediated transient expression of DIR1 or DIR1-like suggest that DIR1 and DIR1-like move to distant leaves during the induction stage of SAR. However, it was still not possible to differentiate DIR1 from DIR1-like in these experiments as the DIR1 antibody recognizes both proteins. To enable future microscopic studies of DIR1-EGFP movement after SAR induction, we chose to generate a stable transgenic line making use of an estrogen inducible promoter (Zuo et al., 2000). We chose this promoter because estrogen-exposed plants exhibit no developmental defects, other chemical inducers are phloem mobile, and the XVE estrogen inducible promoter provides dose-dependent and tightly regulated expression of the gene of interest (Moore et al., 2005).

A number of transgenic lines were created and molecularly characterized to confirm estrogen-specific expression of the transgenes. Before examining DIR1-EGFP translocation from SAR-induced leaves to distant tissues, *in planta* mobility of β-estradiol, the inducer of the XVE promoter, was examined to confirm that it is not mobile. RT-PCR was performed on estrogen-treated lower leaves as well as untreated distant leaves at various time points after estrogen treatment. *DIR1-EGFP* transcripts increased in a time-dependent manner in estrogen-treated leaves and were not detected in distant leaves, indicating that estrogen was not traveling systemically and expression of the transgene was restricted to locally treated leaves (Figure S10A). DIR1-like transcript levels were also examined in distant leaves to ensure that DIR1 antibody signals in distant leaves were not due to up-regulation of DIR1-like during SAR induction. Up-regulation of DIR1-like transcripts was not observed in distant leaves after SAR induction and similar to DIR1 (Figure 3), DIR1-like expression was reduced in SAR-induced leaves compared to untreated leaves (0 hpi) (Figure S10B).

**Figure 8:**
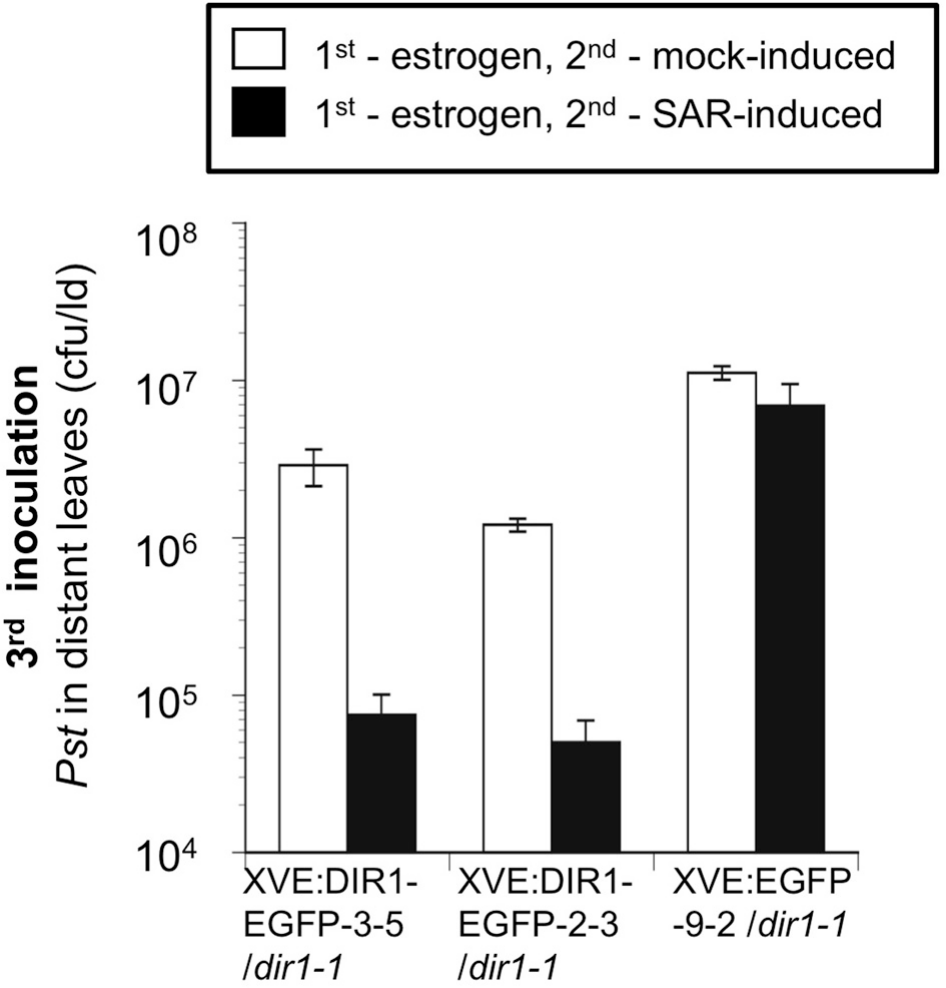
**SAR Assays using estrogen-inducible lines.** EGFP and DIR1-EGFP expression was induced in XVE:DIR1-EGFP/*dir1-1* l(3–5 and 2–3) and XVE:EGFP-9-2/*dir1-1* by infiltrating two lower leaves with estrogen, followed 14–24 h later by a second inoculation to mock- or SAR-induce (*Pst-avrRpt2*) the same leaves. A third inoculation in distant leaves with virulent *Pst* was used to assay for SAR. This experiment was repeated three additional times with similar results.

Expression of DIR1-EGFP was monitored by protein gel blot analysis at various times after estrogen application and SAR induction. A DIR1-EGFP fusion of ~40 kDa, as well as the 7 and 15 kDa DIR1 monomer and dimer-sized bands were observed in leaf extracts using both DIR1 and GFP antibodies (Figure S11). Since DIR1-EGFP and DIR1 were detected in protein gel blots using whole leaf extracts this indicates that the estrogen lines express more DIR1 fusion protein compared to transient expression of DIR1-EYFP by *Agrobacterium*. Two lines, XVE:DIR1-EGFP-2-3/*dir1-1* and XVE:DIR1-EGFP-3-5/*dir1-1* were analyzed in 4 independent estrogen-SAR experiments. DIR1-EGFP expression was induced in two lower leaves by estrogen infiltration, followed 14–24 h later by a second inoculation to mock- or SAR-induce the same leaves. Some plants received a third inoculation in distant leaves with virulent *Pst* to assay for SAR, other plants were used to examine DIR1-EGFP levels in inoculated and distant leaf petiole exudates. When treated with estrogen, both XVE:DIR1-EGFP/*dir1-1* lines displayed a robust SAR response as demonstrated by the 20-fold reduction in *Pst* levels between mock- and SAR-induced plants, while the XVE:EGFP/*dir1-1* control line did not (Figure 8). Estrogen-specific expression of DIR1-EGFP in the induced leaf rescues the SAR deficiency in *dir1-1*.

**Figure 9:**
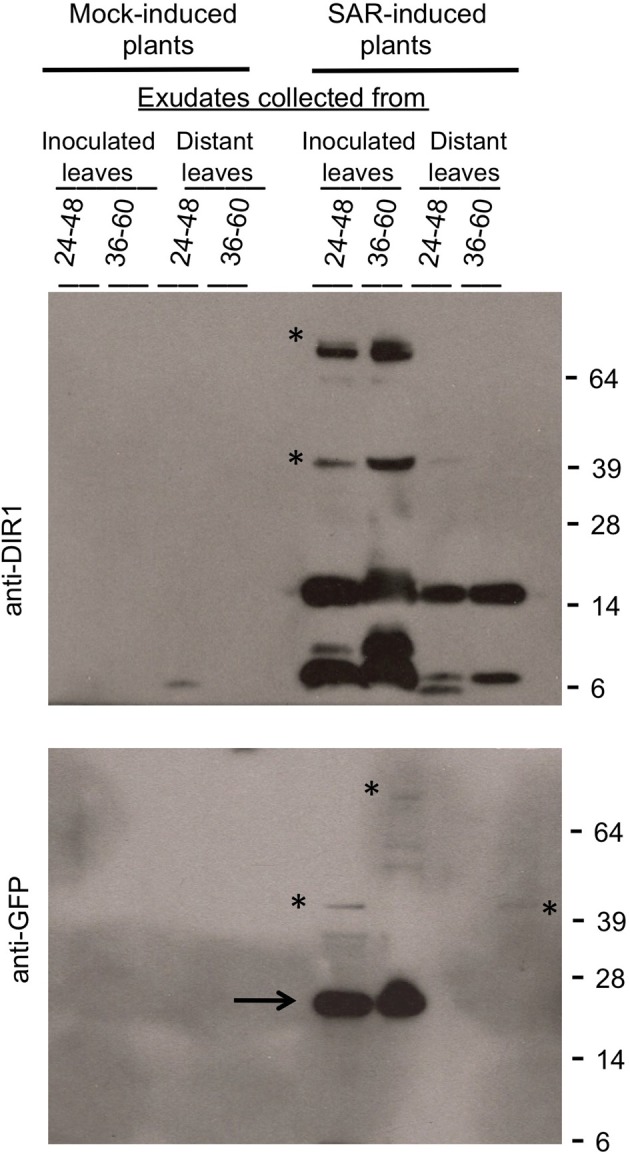
**Detection of DIR1-EGFP in SAR-induced and distant leaf petiole exudates.** Petiole exudates were collected from the experiment shown in Figure 8. DIR1-EGFP expression was induced in the T4 XVE:DIR1-EGFP-2-3/*dir1-1* line by estrogen infiltration, followed by mock- or SAR- induction (*Pst-avrRpt2*) in these same leaves and collection of exudates from inoculated and distant leaves over 24–48 hpi and 36–60 hpi. Exudates were lyophilized and subjected to protein gel blot analysis with the DIR1 (top) or GFP antibodies (bottom). Protein molecular weight markers are indicated (64, 39, 28, 14, 6 kDa). The asterisks indicate signals at ~40 kDa (DIR1-EGFP + DIR1) and at ~66 kDa (2 DIR1-EGFP). The arrow indicates a ~26 kDa EGFP band. This experiment was repeated four times with similar results.

To determine if the DIR1-EGFP fusion is stable and detectable, exudates were concentrated and subjected to protein gel blot analysis using DIR1 and GFP antibodies. Similar to previous gel blots a DIR1 signal was not observed in mock-induced exudates (Figure 9). The DIR1 antibody detected DIR1 monomer- (~7 kDa) and dimer-sized (~15 kDa) bands, as well as ~40 (DIR1-EGFP + DIR1) and ~66 (DIR1-EGFP dimer) kDa bands in exudates collected from estrogen-infiltrated/SAR-induced leaves. A duplicate blot was probed with a GFP antibody and 26 (EGFP alone), 40 and 66 kDa bands were detected in exudates collected from estrogen-infiltrated/SAR induced leaves (Figure 8). Distant leaf exudates collected from estrogen/SAR-induced plants contained DIR1 monomer- and dimer-sized bands and a faint 40 kDa band (DIR1-EGFP + DIR1). These data indicate that DIR1-EGFP fusion proteins of 40 and 66 kDa can be detected using both the DIR1 and GFP antibodies in exudates collected from induced leaves, whereas estrogen/SAR-induced distant petiole exudates contained a faint 40 kDa band plus abundant DIR1 monomer and dimer-sized bands. The XVE:DIR1-EGFP/*dir1-1* lines make it possible to conclude that DIR1-EGFP proteins are moving from the induced leaf down the petiole to distant leaf petioles during the induction and long distance signal movement stages of SAR.

### Using the SAR index to compare agro-SAR and estrogen-SAR assays

The outcome of a plant-pathogen interaction is dependent on the genotypes of the plant and microbe, with environmental conditions comprising the third side of the “disease triangle” (Agrios, 2005). In our experience SAR is highly affected by external environmental conditions even in growth chambers, therefore we perform SAR experiments over a number of years to obtain a more complete picture of the SAR response in the ecotypes or mutants we study. This led us to observe the infrequent (6.7%) and partially SAR-competent phenotype of *dir1-1* and led us to investigate DIR1-like which required that we perform numerous SAR assays over the last 5 years (′07–′11) using two approaches (transient DIR1 and DIR1-like expression and stable inducible DIR1 expression). To objectively compare 5 years of data and two experimental types, a method to quantify the SAR response was needed. First, a SAR response scale (0 to ++++) was created (Table S1) and the SAR^+^ responses per year or across all years were calculated. This provided an overall view of SAR competence as all SAR responses from modest (+) to robust (++++) were considered to be SAR^+^ such that transient expression of DIR1-like and DIR1-EYFP in *dir1-1* had similar SAR^+^ responses over all years (71 and 75%, respectively). Next, a SAR index was created to account for varying degrees of SAR competence and varying numbers of experiments and is defined as the sum of (SAR response x # experiments)/total # experiments for each year or average for all years (Table S1). Stable expression of DIR1-EGFP and EGFP (endogenous DIR1-like) from the estrogen-inducible XVE promoter in the *dir1-1* mutant had SAR indices of 4 and 0, respectively, compared to 1.5 for DIR1-EYFP, 1.0 for DIR1-like and 0.4 for endogenous DIR1-like (EYFP control) in Agro-SAR assays, indicating that the XVE:DIR1-EGFP/*dir1-1* stably transformed line is highly SAR-competent. SAR index values were also compared to examine the contribution of endogenous DIR1-like (EYFP control) to DIR1-like expressed transiently from the 35S promoter, such that *dir1-1* expressing endogenous DIR1-like had a lower SAR index of 0.4, compared to 1.0 for transiently expressed DIR1-like. Although DIR1-like expressed transiently by *Agrobacterium* rescued the *dir1-1* defect with a SAR index of 1.0, transiently expressed DIR1-EYFP had a higher SAR index of 1.5. Therefore, the SAR index is a valuable tool that provides a quantitative way to compare numerous assays performed over many years and provides support for the idea that DIR1-like (expressed from its endogenous promoter or transiently from the 35S promoter) contributes less effectively to SAR compared to DIR1.

## Discussion

The occasionally SAR-competent phenotype of *dir1-1* led us to investigate the role of DIR1-like, a highly similar gene adjacent to DIR1 on chromosome 5. Our phylogenetic analysis provides evidence that DIR1 and DIR1-like are paralogous genes and that this duplication occurred before the divergence of *A. thaliana* and *A. lyrata*. We developed an *Agrobacterium*-mediated transient transformation/SAR assay to examine DIR1 movement during SAR and to examine DIR1-like's participation in SAR. It was possible to rescue the SAR defect in *dir1-1* by transiently expressing DIR1 in one leaf followed by SAR induction in the same leaf, demonstrating the utility of the Agro-SAR assay and providing evidence that transient expression of DIR1 in a single induced leaf is sufficient to rescue the SAR defect in *dir1-1*. Given that DIR1-like is expressed at wild-type levels in *dir1-1* and *dir1-1* is rarely SAR-competent, it was surprising that *Agrobacterium*-mediated transient expression of DIR1-like rescued the SAR-defect in *dir1-1* in 3 of 4 experiments. Because *Agrobacterium*-mediated transient expression does not support high levels of ectopic expression in *Arabidopsis* (Tsuda et al., 2012 and this work), we propose that during the Agro-SAR assay, sufficient *DIR1-like* expression occurs in leaf cells particularly important for SAR induction resulting in rescue of the *dir1-1* SAR defect.

Transient expression of DIR1 or DIR1-like did not rescue the SAR defect in the *npr1-2* SAR mutant, supporting the idea that NPR1 acts downstream of DIR1 in distant leaves during SAR [discussed in Champigny and Cameron (2009)]. Moreover, a DIR1-sized band was detected in *npr1-2* exudates collected from leaves induced for SAR suggesting that DIR1 long distance movement does not require the presence of NPR1.

DIR1 protein was typically undetectable in leaf extracts obtained from wild-type and DIR1 promoter transgenic lines and could be detected only in concentrated petiole exudates or IWFs suggesting that endogenous DIR1 levels are low in *Arabidopsis* (Champigny et al., 2011 and this work). We also demonstrated that expression of DIR1 and DIR1-like is further reduced during SAR induction (Champigny et al., 2011, this work), reinforcing the idea that only a small amount of DIR1 is required to manifest the SAR response. Using a proteomics-based approach, we attempted to identify DIR1 and/or DIR1-like in SAR-induced petiole exudates. However, neither DIR1 nor DIR1-like protein could be detected by LC-MS/MS (liquid chromatography followed by tandem mass spectrometry) even in pooled, concentrated exudates collected from 35S:DIR1 overexpression lines. DIR1 moving into petiole exudates collected from wild-type plants and our transgenic lines can only be detected using highly sensitive chemiluminescent protein gel blot analysis (Pierce—pico to femtogram detection range). Based on all of these observations, the amount of DIR1 contributing to long distance signaling in wild-type plants appears to be very low.

*Agrobacterium*-mediated transient expression in Arabidopsis is rarely reported in the literature (Nimchuk et al., 2000; Wroblewski et al., 2005; Tsuda et al., 2012) as insufficient expression of the gene of interest is observed, probably due to an effective basal/PAMP-triggered immune response initiated by *Arabidopsis* to *Agrobacterium* (Zipfel et al., 2006). Nevertheless, transient expression of DIR1 and DIR1-like in single leaves using this method was sufficient to rescue the SAR defect in *dir1-1*, likely due to the fact that little DIR1 is necessary for SAR as discussed above. The Agro-SAR assay offers some advantages over transgenic approaches, chiefly the time required to generate and characterize transgenic lines, and can be employed to test the role of newly discovered genes in the SAR response. For example, our experiments establish that expression of DIR1 is required only within SAR-induced leaves. The Agro-SAR assay could be used to test the spatial and temporal requirements for other SAR proteins during the induction or manifestation stages of SAR.

Unexpected results were obtained when protein blots were performed on petiole exudates and IWFs collected from plants expressing DIR1-GUS or DIR1-EYFP. IWFs as well as petiole exudates collected from these plants consistently exhibited a ~15 and sometimes ~7 kDa signal, but not the 75 or 33 kDa signals expected of DIR1-GUS or DIR1-EYFP fusion proteins, respectively. We considered but ruled out the possibility that the fusion proteins were not expressed because plants expressing DIR1-fusion proteins were capable of rescuing the *dir1-1* SAR defect. Secondly, the expected 75 kDa DIR1-GUS fusion was detected in whole leaves from DIR1-GUS transgenics using both DIR1 and GUS antibodies. As expected, transgenics expressing GUS fusions with either full-length DIR1 or DIR1 lacking its signal sequence exhibited intracellular GUS activity in whole leaves and leaf cross-sections. Additionally, IWFs isolated from DIR1-GUS transgenics contained an abundant ~15 kDa DIR1 signal and GUS enzyme activity, indicating that DIR1 and GUS coexist as active proteins within the apoplast of these plants. Our results suggest that GUS and EYFP are cleaved from DIR1 such that little DIR1 fusion protein is present in the apoplast or phloem sap-enriched petiole exudates. Fortunately, it was possible to detect DIR1-EGFP expressed from the estrogen-inducible promoter in exudates collected from SAR-induced leaves. However, a fraction of the DIR1-EGFP fusion proteins were cleaved such that DIR1 monomer/dimer and EGFP monomer-sized bands were observed. In distant leaf exudates, DIR1 monomer- (7 kDa) and dimer-sized (15 kDa) bands were abundantly present, while only a modest signal of DIR1-EGFP (~40 kDa) was detected indicating that many fusion molecules were cleaved during movement to distant leaves. Using both the DIR1 and GFP antibodies, ~40 and ~66 kDa bands were detected in exudates from SAR-induced leaves, however the GFP antibody signal was less intense than the DIR1 antibody signal. Although caution must be used when comparing band intensities of independent blots using different antibodies, an alternative explanation for the ~40 and ~66 kDa DIR1 antibody bands, is that DIR1 monomers associated with other proteins in a DTT-resistant manner, given that 200 mM DTT was added to the exudates before electrophoresis and blotting. Either explanation (DIR1 dimers or DIR1-protein complexes) fits with the appearance of trypsin-sensitive high molecular weight fractions in dehydroabietinal-elicited SAR observed by Chaturvedi et al. (2012). Nevertheless, DIR1-EGFP-sized bands (~40 and ~66 kDa) were detected with the GFP antibody in exudates collected from both SAR-induced and distant leaves, providing evidence that DIR1-EGFP moved from SAR-induced to distant leaves.

Cleavage of peptide moieties from fusion proteins expressed in plants is infrequently documented in the literature. In one report (daSilva et al., 2006), it was observed that a translational fusion between GFP and a vacuolar protein was mislocated to the apoplast and resulted in cleavage of GFP in transgenic tobacco. In addition, expression of an HA- and GFP-tagged Cf-9 transmembrane resistance receptor, in yeast, *Arabidopsis* and tobacco cells resulted in appropriate accumulation of the fusion protein within the vacuole of yeast cells but inappropriate secretion to the apoplast followed by cleavage of the HA and GFP tags in both plants (Benghezal et al., 2000). It is interesting to note that in this study, unexpected cleavage of DIR1 fusion proteins appears to occur during movement of DIR1 to distant leaves via the phloem and in the apoplast (IWFs), which is consistent with numerous observations that the apoplast is enriched in proteases (Misas-Villamil and Al van der Hoorn, 2008).

The movement of native DIR1 in SAR-induced *Arabidopsis* was not addressed in our previous reports (Maldonado et al., 2002; Champigny et al., 2011) or in Chanda et al. (2011) although numerous reviews (Durrant and Dong, 2004; Parker, 2009; Dempsey and Klessig, 2012) present models in which DIR1 translocates to distant tissues during SAR. Because petiole exudates collected from *dir1-1* plants did not induce SAR marker (PR1) expression in distant leaves, the data presented in Maldonado et al. (2002) supports a role for DIR1 in the production or long-distance translocation, of a SAR signal. Chanda et al. (2011) observed the accumulation of ectopically expressed *Arabidopsis* DIR1-EGFP in *N. benthamiana* distant leaves after exogenous G3P treatment. Therefore, the data presented here fills a gap in the SAR model by providing compelling evidence for the long-distance translocation of DIR1 during biologically-induced SAR in *Arabidopsis*.

DIR1 antibody signals of both ~7 and 15 kDa were detected in protein gel blot analysis of petiole exudates and IWFs collected from wild type plants suggesting that DIR1 is present in monomeric form and also exists as either a homodimer as is the case for the peach Prup3 LTP (Pasquato et al., 2005) or as a heterodimer with another LTP, as is the case for the barley LTP1-LTP2 complex (Gorjanović et al., 2005; Gorjanović, 2007). The LTP1-LTP2 dimer was disrupted in the presence of reducing agents suggesting that disulfide bonds hold the monomers together (Gorjanović, 2007). Incubation of DIR1 exudates in elevated concentrations of a reducing agent (200 mM) resulted in the appearance of the ~7 kDa band. These experiments suggest that DIR1 participates in dimers in which the monomers are held together by DTT-resistant interactions. Although we do not preclude that DIR1 could participate in a heterodimer with DIR1-like or another protein, protein gel blots performed on petiole exudates collected from the SAR induced XVE:DIR1-EGFP lines suggest that DIR1 dimerizes with itself. In these experiments 7 kD and 15 kD signals were detected along with a 66 kDa signal which is the predicted size of a homodimer comprised of two DIR1-EGFP molecules.

In our studies using wild-type Ws and DIR1pro:DIR1-GUS/*dir1-1* lines, faint DIR1-sized bands were detected in petiole exudates collected between 14 and 20 h post SAR induction, with band intensity increasing from 38 to 44 hpi suggesting that it takes the SAR signal at least 14 h to move down the petiole. However, leaf detachment experiments demonstrated that it takes 4 h in cucumber (Rasmussen et al., 1991) and 4–6 h in *Arabidopsis* (Truman et al., 2007; Chaturvedi et al., 2012) for the SAR signal to move out of the induced leaf. It may be that DIR1 does move out of the induced leaf between 4 and 6 h post SAR induction, but at levels below the detection limit of protein gel blot analysis until sufficient DIR1 accumulates in petiole exudates by 14–20 h post SAR-induction.

To distinguish the roles of DIR1 and DIR1-like during SAR, transgenic plants expressing 35S:antisenseDIR1-like in Ws and the *dir1-1* mutant were created to generate *dir1-like* and *dir1dir1-like* double mutants, respectively. Although potential *dir1-like* single and *dir1dir1-like* double mutants were identified in over 50 transgenic lines (T2 generation) using qRT-PCR (data not shown), homozygous T3 plant lines with reduced DIR1-like expression were not recovered. We observed that *DIR1-like* expression in the T3 35S:antisenseDIR1-like lines was not silenced, instead *DIR1-like* was expressed at wild-type levels. In addition, several T2 35S:antisenseDIR1-like/*dir1-1* lines with reduced expression of *DIR1-like* failed to produce viable seeds suggesting that reduction of *DIR1-like* expression is lethal. These observations suggest that DIR1-like function may be important during seed development. Estrogen-inducible DIR1-like RNAi lines will be created to circumvent the lethality issue and further investigate DIR1-like's contribution to SAR.

Since the discovery of DIR1 and its involvement in SAR long distance signaling (Maldonado et al., 2002), a number of studies report the existence of multiple SAR mobile signals, including MeSA (Park et al., 2007; Vlot et al., 2008; Liu et al., 2010b), jasmonic acid (JA) (Truman et al., 2007), azeleic acid (AA) (Jung et al., 2009), a CDR1-derived peptide (Xia et al., 2004), plastid glycerolipids (Nandi et al., 2004; Chaturvedi et al., 2008), G3P (Chanda et al., 2011) and an abietane diterpenoid, dehydroabietinal (Chaturvedi et al., 2012). However, two studies suggest that JA (Chaturvedi et al., 2008; Attaran et al., 2009) and MeSA (Attaran et al., 2009) are not SAR long distance signals. It has been postulated that multiple mobile signals participate in SAR long distance signaling and that environmental conditions, developmental plant age and the particular plant-pathogen system may impact which signals are required and this may explain what appear to be contradictory results (Champigny and Cameron, 2009; Liu et al., 2010b). In this work, we provide evidence suggesting that DIR1-like occasionally contributes to SAR. This may explain the variable results observed in different labs [discussed in Dempsey and Klessig (2012)]. For example differential SAR responses are observed when plants receive different amounts of light after SAR induction in both wild type and SAR mutants including *dir1-1* (Liu et al., 2011b). However, it is still possible that the partial SAR response occasionally observed in *dir1-1* is not due to DIR1-like.

Using G3P infiltration assays, Chanda et al. (2011) suggest that DIR1 is required for G3P-induced resistance and investigated the effect of G3P on DIR1 movement. After infiltration of ^14^C-G3P and recombinant DIR1 into Arabidopsis leaves, ^14^C-G3P was detected in distant leaves. From this, Chanda et al. (2011) suggested that DIR1 is required for G3P movement during SAR. However, a high level of DIR1 protein (20 ug) was infiltrated into leaves perhaps inducing necrosis which in turn could induce G3P movement. Moreover, it is unlikely that DIR1's four disulphide bonds formed correctly during expression in the *E. coli* expression system used. Therefore, the role of G3P in DIR1 movement requires further investigation. Chaturvedi et al. (2008) suggest that DIR1 and plastid glycerolipids act together during long distance signaling. Moreover, DIR1 is required for AA-induced (Jung et al., 2009) and dehydroabietinal-induced resistance (Chaturvedi et al., 2012). These studies along with the DIR1 movement data presented in this work, lead us to hypothesize that DIR1 may interact with one or more of these molecules acting as a chaperone or component of a complex(s) that moves to distant leaves during SAR.

## Materials and methods

### Plant growth conditions

*Arabidopsis* seeds from wild type (ecotype Ws-2), *dir1-1, npr1-2* and all transgenic *Arabidopsis* lines were surface sterilized and germinated on solid Murashige and Skoog (MS) medium for 5–7 days under continuous light. Seedlings were transferred to soil (Sunshine Mix #1), hydrated with 1 g/L 20-20-20 fertilizer and grown for 3–4 weeks at 22°C, 9 h photoperiod at 150 μE m^−2^ s^−1^ light intensity and 65–85% relative humidity.

### Pathogen culture and inoculation

Virulent (containing pVSP1) and avirulent (containing pVSP1 + *avrRpt2*) *Pseudomonas syringae* pv. *tomato* DC3000 strains used in this study are previously described (Whalen et al., 1991). SAR experiments at McMaster were sometimes done using the coronatine mutant *Pseudomonas syringae* pv *maculicola* ES4326 (*Psm*) containing *avrRpt2* strain (Cui et al., 2005). No difference was found between these strains in terms of their ability to induce SAR. Bacteria were cultured overnight in King's B medium, diluted to either 10^5^ or 10^6^ cfu ml^−1^ in 10 mM MgCl_2_ and pressure infiltrated into the abaxial side of a leaf using a needleless 1 ml syringe. Quantification of *in planta* bacterial levels was performed by dilution plating essentially as described (Cameron et al., 1999). *Agrobacterium tumefaciens* strain GV3101 (Agro) was cultured as described in Wroblewski et al. (2005).

### Agro-SAR and estrogen-SAR assays

Agro-SAR and estrogen-SAR assays were performed on 3.5–4 weeks old plants (24–28 days post germination, dpg). All SAR assays were performed between June and October as SAR assays are rarely successful during the winter and spring even in wild type Ws-2 plants. Plants were SAR-induced by inoculation of two lower leaves with avirulent *Pst (avrRpt2)* (10^6^ cfu ml^−1^) or mock-inoculated, followed by challenge inoculation of distant leaves with 10^5^ cfu ml^−1^ virulent *Pst* DC3000 and *in planta* bacterial level determination 3 dpi. Agro-SAR experiments were conducted by transiently expressing DIR1:EYFP or DIR1-like in two lower leaves by inoculation with *Agrobacterium tumefaciens* GV3101 (0.4 OD_600_) harboring *35S:DIR1-EYFP* or *35S:DIR1-like* binary expression vectors (Wroblewski et al., 2005). At 4 dpi, agro-inoculated leaves were then mock- or SAR-induced followed by challenge inoculation with virulent *Pst* in distant leaves as described above. Estrogen-SAR assays were performed by first pressure infiltrating two lower leaves of XVE:DIR1-EGFP/*dir1-1* or XVE:EGFP*/dir1-1* lines with 50 μM β-estradiol to initiate expression of the transgene. 14–24 h following estrogen treatment, the same lower leaves were mock- or SAR-induced followed by challenge inoculation with virulent *Pst* in distant leaves 3 days later as described above. The first stage of SAR, induction with *Pst (avrRpt2)* was always performed in the morning between 9 am and noon, for all SAR assays.

### Intercellular washing fluid, petiole exudate collection and leaf protein extractions

Fully expanded leaves of 3–4 weeks old *Arabidopsis* plants were vacuum infiltrated with sterile distilled water for 30 min, blotted with absorbent paper to dry the leaf surfaces, followed by IWF collection from leaves by centrifugation at 1000*g* for 30 min at 4°C (Kus et al., 2002). Fifty leaves produced approximately 200–300 μl IWF. IWFs were sampled immediately for GUS activity or frozen at −20°C until protein gel blot analysis was performed.

The *Arabidopsis* petiole exudate method was modified from the method of King and Zeevaart (1974). Leaves were mock-inoculated or induced for SAR by inoculation with *Pst (avrRpt2)* (10^6^ cfu ml^−1^). By using a lower dose (10^6^ cfu ml^−1^), not all cells in the leaf undergo the HR, therefore the leaf and petioles remain intact throughout the exudation process. Petioles exudates were collected at different times after induction by cutting one petiole at a time just above the stem, followed by surface sterilization for 10 s (50% ethanol, 0.0006% bleach), rinsing in sterile 1 mM EDTA and submerging petioles in ~1.5 ml 1 mM EDTA (must be done quickly to prevent sieve element clogging) and 50 μg ml^−1^ ampicillin (to kill any remaining surface bacteria). The sterilization step must be long enough to kill *Pst* clinging to leaf surfaces, but not long enough to kill the *Pst* in the plant intercellular spaces. The petioles of seven to ten leaves (depending on size) per microfuge tube were allowed to exude phloem sap over 2 days (early experiments) in a humid environment (90–100%) on the lab bench, to produce one exudate. In later experiments, petioles were allowed to exude for less time (5 or 24 h) to reduce leakage of proteins from petiole cells that occurs because of EDTA-induced tissue softening (Hepler, 2005). Petiole exudate samples (~1.5 ml) contained between 5 and 50 μg total protein (Biorad Protein Assay Kit) and were stored at −20°C until concentration by lyophilzation followed by protein gel blot analysis. Leaf weight could not be used to normalize exudate amounts loaded per lane as cut petioles must be immersed immediately in EDTA, allowing no time for weighing. In some experiments, exudate total protein levels were in the lower range (3–10 ug/exudate) and a DIR1 antibody signal could still be detected. Therefore, for each experiment, the same number of exudates per lane were loaded (1–4) to detect a DIR1 antibody signal.

Total protein extracts were prepared from leaf tissue by grinding flash-frozen leaves in liquid nitrogen to a fine powder and extracting with 50 mM Tris pH 7.5, 1 mM EDTA, 100 mM NaCl, 1% NP-40, 0.1% SDS, 0.1% Triton X-100, 0.7% 2-mercaptoethanol, 1mM PMSF. Protein concentration was determined by Bradford assay (Biorad Protein Assay Kit) and 30–50 μg was loaded per lane.

### DIR1 transcript abundance determination using RT-PCR

Total RNA was isolated from frozen leaf samples from various Arabidopsis transgenic lines, 35S:DIR1-5E/dir1-1, DIR1pro:DIR1:GUS-29/dir1-1, 35Spro:antisenseDIR1-3B, and in addition to *dir1-1* and wild-type Ws plants, using Trizol reagent (Invitrogen, Carlsbad, CA). Two micrograms of the total RNA was treated with RNase-free DNase I (Invitrogen, life technologies) as per the manufacturer's instructions. RT-PCR of DIR1 expression with DNase I-treated RNA (500 ng) was performed using One-Step RT-PCR kit (Qiagen) and a primer pair (5′-ATGGCGACGAAGAAAGCAGC-3′ and 5′-AACAATTGGGGCGTTGGCTAG-3′) designed from coding sequence. All RT-PCR reactions were performed with 2 μl of RT-PCR enzyme mix, the buffer provided by the supplier, 0.2 mM dNTPs, and a primer pair (0.6 μM each) in a final volume of 25 μl. The RT-PCR conditions were as follows: 50°C for 30 min and 95°C for 15 min for RT steps followed by 28 PCR cycle of 94°C for 30 s, 60°C for 45 s, and 72°C for 1 min, with a final polymerization step at 72°C for 10 min. Actin 1 (At2g37620) was used as control for RNA levels in each sample using primers Actin1F (5′-GGCGATGAAGCTCAATCCAAACG-3′) and Actin1R (5′-GGTCACGACCAGCAAGATCAAGACG-3′). DIR1-EYFP expression was monitored using RT-PCR (same conditions as above) in leaves inoculated with Agrobacterium (35S: DIR1-EYFP) at 1, 2, 3, and 4 dpi using forward DYfusion5p (5′-GGTGTTGATCCTGAACTCGC-3′) and reverse primers, DYfusion3p (5′-AACTTCAGGGTCAGCTTGCC-3′). These primers produce a PCR product that spans the DIR1 EYFP fusion.

### Quantitative RT-PCR (absolute quantification method—fobert lab)

Flash frozen leaf tissue (0.1–0.2 g) was ground to a fine powder in liquid nitrogen and total RNA was extracted according to the manufacturer's instructions with a Plant Mini RNA isolation kit (Qiagen). RNA was treated with DNAse I (Invitrogen) to remove contaminating genomic DNA then 2 μg treated RNA was reverse transcribed to cDNA with Superscript II (Invitrogen) polymerase. The cDNA was diluted 10-fold with water and subject to kinetic PCR amplification on a MX3000P spectrofluorometric thermal cycler (Stratagene). Total volume reactions were of 12.5 μL and contained 5 ng cDNA and 1 X SYBR Green® (Quantitech, Qiagen). The annealing and elongation step of the amplification cycle was performed at 66°C and 40 cycles were used. Primers used to amplify *DIR1* transcript were: Forward primer 5′-GATCGTGATAATGGCTATGTTGGTCGATACATC, reverse primer 5′-GCGTTGGCTAGACCACACTGTTTGGGGAGAGC. Primers used to amplify *DIR1*-like (At5g48490) transcript were: forward primer 5′-AATGGTGATGGCTAGTTTAGTCGTTGAGAGG, reverse primer 5′-TAAACAAACAAAGGAAAACACCATAATGC. Specificity of PCR primers was verified by sequencing of representative PCR products. The number of transcript molecules was calculated using the “absolute quantification via Ct” method (Rutledge and Stewart, 2008). Gene expression was standardized to expression levels of *UBQ5*, whose transcripts were monitored with forward primer 5′-AGCTTACAAAATTCCCAAATAGAAATGCAG and reverse primer 5′-ACCTACGTTTACCAGAAAGAAGGAGTTGAA.

### Quantitative RT-PCR (absolute quantitation method—cameron lab)

Total RNA was extracted from Arabidopsis leaf tissue using Trizol (sigma) method followed by DNA removal using Turbo DNase Free (Ambion). First strand cDNA synthesis was performed using 2 μg of total RNA as template which was reverse transcribed into cDNA by MMLV (Life Science Technologies) using manufacturer instructions and diluted 3 fold in water before use. qRT-PCR was performed in a 10 ul reaction consisting of 2 ul of diluted cDNA, LuminoCT SYBR Green qPCR ready mix (Sigma) and 200 or 400nM of primer. The mixture was loaded into low profile optical 96-well plates. qRT-PCR was performed in the Bio-Rad CFX96 touch™ Real-Time PCR Detection System and analyzed using BioRad CFX manager 2.0 software. Gene specific primers were validated for specificity and efficiency using an 8 point standard curve and purified products were Sanger sequenced to confirm identity. Primer secondary structure was evaluated using mfold↓ (http://mfold.rna.albany.edu/). Primers used were 5′ TCGTGATAATGGCTATGTTGGTC 3′ and 5′ ACTGTTTGGGGAGAGCAGAAG 3′ for DIR1, 5′ AATAAAGAGGATAAAATGACAAGC 3′ and 5′ CTGGTAAGCATTCATTCAACTC 3′ for DIR1-like, 5′ TGTCCGCAAATCCCTAAAAG 3′ and 5′ CCAGGGAGCTTCAAGAACAG 3′ for 5FCL. Genevestigator was used to choose the 5FCL reference gene based on the transcript stability and a similar expression range to our genes of interest. All samples were analyzed in three biological and technical replicates. A no template control as well as a no reverse transcriptase control were run simultaneously with the samples. For absolute quantification, standard curves (# of template copies vs. *C_T_* plots) generated from known quantities of DNA templates were used to convert real-time qRT-PCR data into absolute (Lu et al., 2012). PCR generated templates were separated on an agarose gel and purified. Fluorometry (Promega QuantiFluor™ dsDNA system) was then used to determine the exact concentration in copies/μl of the templates. For each gene product a standard curve was constructed using a 10-fold serial dilution series ranging from 1 to 1 × 10^8^ copies/μL of DNA template. Absolute values were then calculated using the standard curve equation of the line. To account for variations in starting RNA template these final absolute values in number of transcripts/ng of RNA were divided by the number of transcripts/ng of RNA of the reference gene 5FCL.

### Protein gel blot analysis

Protein samples (total protein lysates, IWFs or petiole exudates concentrated by lyophilization) were mixed with 5X SDS loading buffer (350 mM Tris-HCL pH 6.8, 30% glycerol, 10% SDS, 0.01% bromophenol blue and 0, 5, 200 mM DTT, followed by boiling for 5 min. 2 to 4 individual lyophilized exudates were reconstituted in loading buffer (5 mM DTT in Figures 1, 2, Figures S1–S3; 200 mM DTT in Figures 5–7, 9 and Figures S6, S9, S11, S12) and loaded per lane. Samples were loaded onto 4–12% NuPAGE Bis-Tris polyacrylamide gels (Invitrogen) and subjected to electrophoresis in MES running buffer. Proteins were transferred to nitrocellulose membranes (Schleicher and Schuel) in Towbin transfer buffer (25 mM Tris base, 192 mM glycine, 20% methanol). Membranes were probed either with anti-DIR1 antisera (Maldonado et al., 2002) at a 1:20,000 dilution, anti-EGFP antibody (Clontech 63259) at 1:10000 dilution or anti-GUS antibody (Molecular Probes A5790) at 1:10,000 dilution in 5% non-fat milk in TBST. Antibody binding was detected with a goat anti-rabbit horseradish peroxidase conjugate and WestFemto reagents (Pierce) as described by the manufacturer.

### Bioinformatics

Coding sequence and amino acid sequences of DIR1 (AT5G48485) and DIR1-like (AT5G48490) were retrieved from The *Arabidopsis* Information Resource (http://www.arabidopsis.org). Sequences were compared using the EMBOSS pairwise alignment algorithm (http://www.ebi.ac.uk/emboss/align). Signal peptides were deduced using the SignalP 3.0 prediction server at www.cbs.dtu.dk/services/SignalP. A SWISS-MODEL homology model of DIR1-like was produced using the Lascombe et al. (2008) DIR1-phosopholipid crystal structure as a template (Peitsch, 1995; Schwede et al., 2003; Arnold et al., 2006; Kiefer et al., 2009). The Swiss-pdf viewer 4.0.1 was used to compare the DIR1 structure and the DIR1-like protein model [http:/www.expasy.org/spdbv/] (Guex and Peitsch, 1997).

### Phylogenetic analyses

A rooted phylogenetic Maximum Likelihood tree of DIR1 and DIR1-like proteins was created using protein sequences lacking the divergent ER signal sequence. Signal P 4.0 was used to determine where the signal sequence cleavage site was located (Perterson et al., 2011). The sequences were aligned in MEGA 5 using Muscle (Tamura et al., 2011). The evolutionary history was inferred using the Maximum Likelihood method based on the Kimura 2-parameter (Kimura, 1980) model with discrete Gamma distribution using MEGA 5 (Tamura et al., 2011). 10,000 bootstrap replicates were conducted and percent bootstrap values were placed on the branches (Felsenstein, 1985). Branches were drawn to scale, measured in number of substitutions per site and were labeled by species name followed by TAIR gene number or Phytozome 8.0 accession.

## See supplementary methods for

Construction of *Agrobacterium* strains and XVE:DIR1-EGFP transgenic lines and Recombinant protein production and DIR1-antibody specificity testing.

## Author contributions

Robin K. Cameron designed and orchestrated the research, performed a number of the petiole exudate/protein gel blot experiments and wrote most of the manuscript. All lab members performed petiole exudate experiments. Jennifer Faubert conceived of the distant leaf petiole exudate experiments, Marisa Isaacs conceived of and performed the DIR1-like homology modeling and DIR1/DIR1-like phylogeny. Marc J. Champigny contributed significantly to writing the manuscript, constructed the *Agrobacterium* 35S:DIR1-EYFP, DIR1-like, and EYFP lines and conceived of and constructed the transgenic XVE:DIR1-EGFP lines (Fobert Lab). Philip Carella and Marisa Isaacs conceived of and performed the experiments to test the specificity of the DIR1 antibody. Absolute quantitative RT-PCR experiments were performed by Marc J. Champigny and Jennifer Faubert in the Fobert lab and by Marisa Isaacs and Philip Carella in the Cameron lab.

### Conflict of interest statement

The authors declare that the research was conducted in the absence of any commercial or financial relationships that could be construed as a potential conflict of interest.
