# Supplementary material for: Long distance movement of DIR1 and investigation of the role of DIR1-like during systemic acquired resistance in Arabidopsis
Source: Front Plant Sci. 2013 Jul 4;4:230. doi: 10.3389/fpls.2013.00230 (PMC3701462; doi:10.3389/fpls.2013.00230)
Supplement: Supplementary file 1 [file 46838_Cameron_Presentation1.PDF]

SUPPLEMENTARY FIGURES

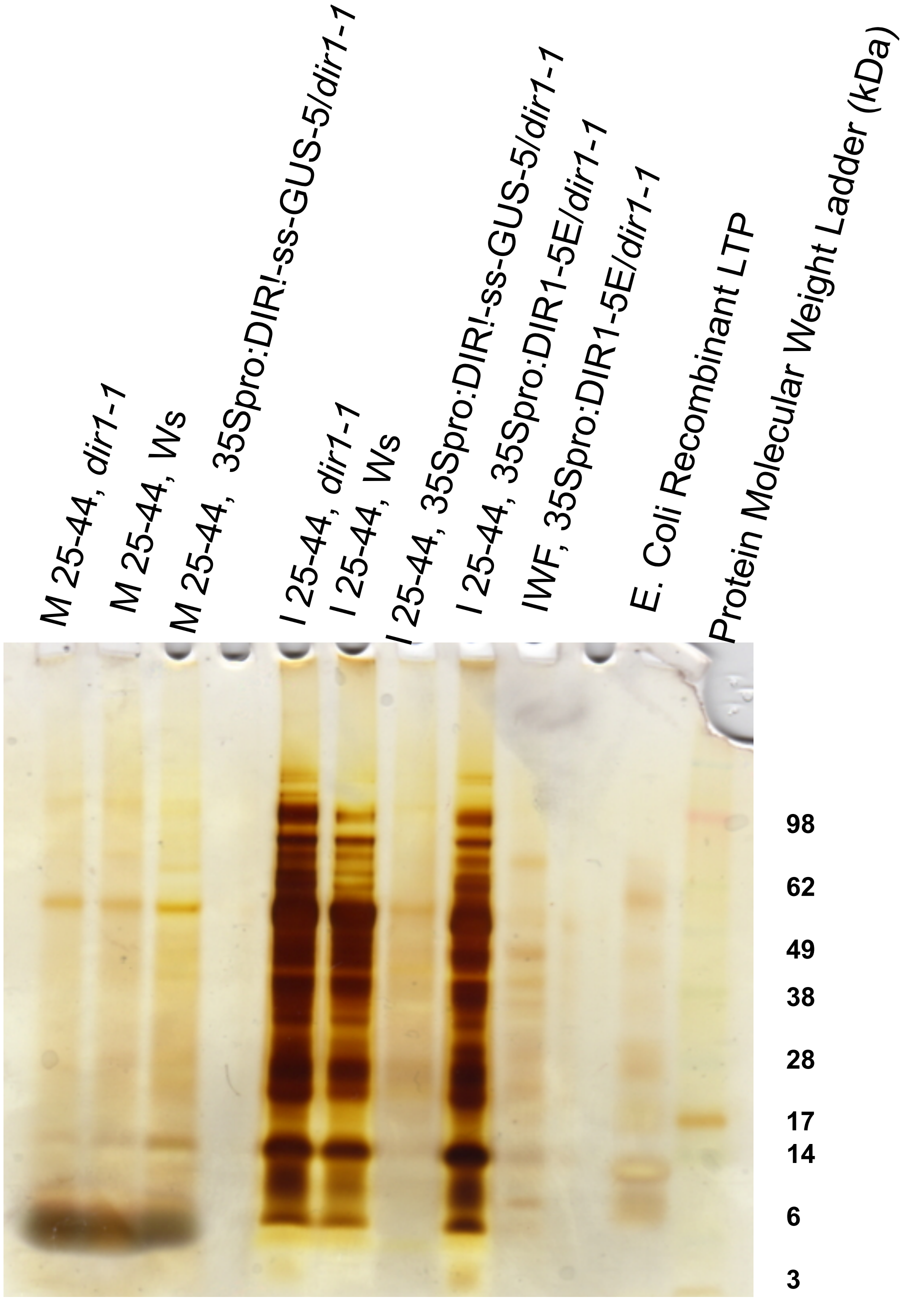

Supplementary Figure 1

Petiole exudates were collected from 25 to 44 hpi from various Arabidopsis plant lines that were either mock-inoculated (M) or SAR-induced (I), lyophilized and subjected to SDS-PAGE and silver stained, 1 exudate per lane (See Methods for details).

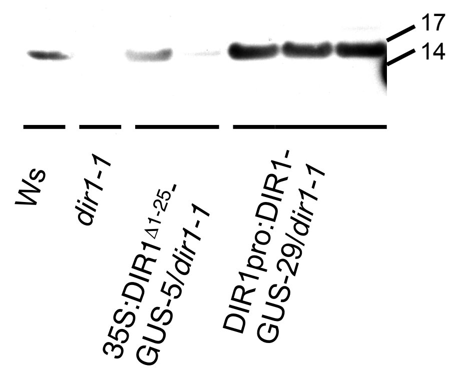

Supplementary Figure 2. DIR1 is detected in intercellular washing fluids (IWFs). IWFs were collected from untreated leaves of Ws, *dir1-1*, 35S:DIR1$^{\Delta 1\text{-}25}$-GUS-5/*dir1-1*, and DIR1pro:DIR1-GUS-29/*dir1-1*. The IWFs were either lyophilized and subjected to protein gel blot analysis. Molecular weight markers (14 and 17 kDa) are indicated. This experiment was repeated 2 additional times with similar results.

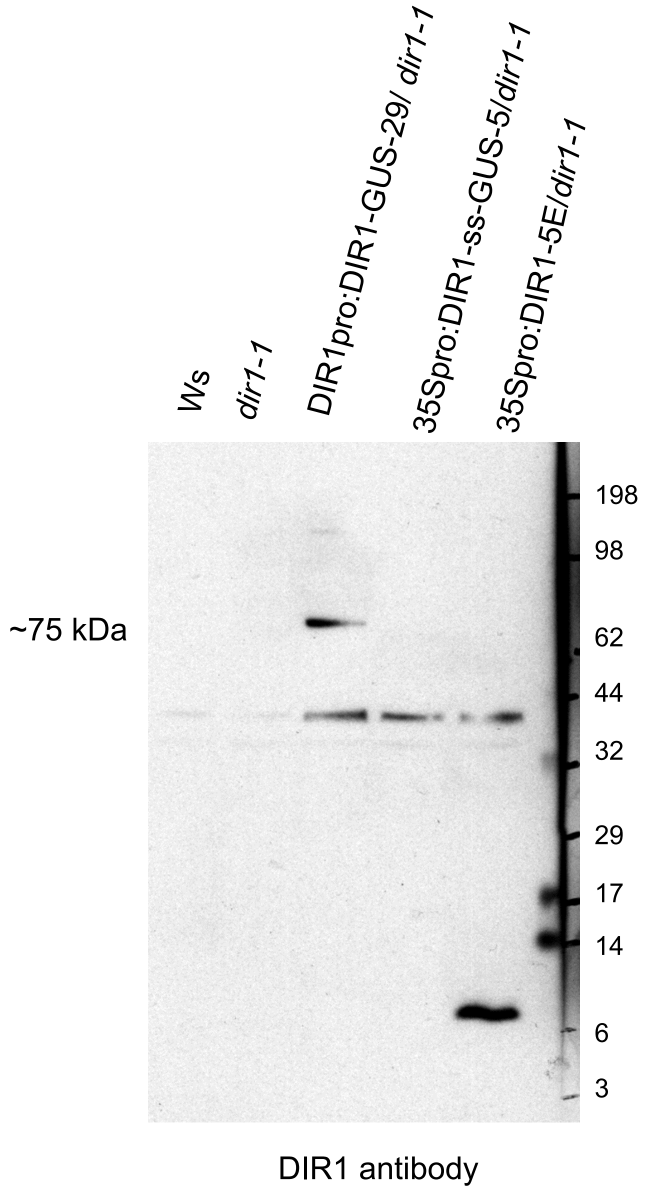

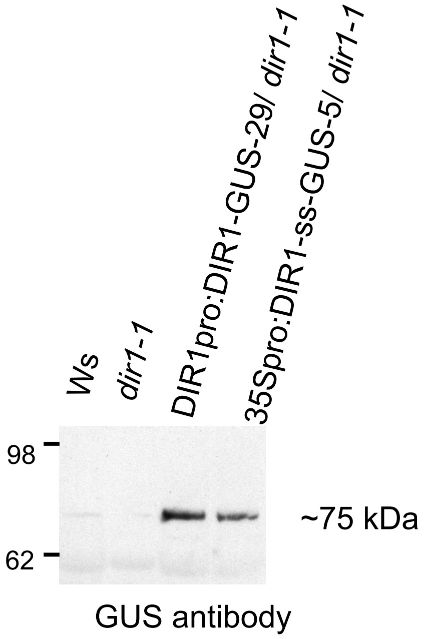

Supplementary Figure 3.

Leaf extracts were collected from various plant lines and subjected to protein gel blot analysis (30 µg total protein per lane). See Supplementary Methods for details. Protein molecular weight markers are indicated.

A

```
DIR1       ATGGCGAGCAAGAAAGCAGCTATGGTTATGATGGCGATGATCGTGATAAT 50 At5g48485
           |||.|.||||||||.|      |||..||.||||.|||..|..||.|.||
DIR1-like  ATGACAAGCAAGAAGG------TGGCAATTATGGTGATTGTAATGGTGAT 44 At5g48490

           GGCTATGTTGGTC---GATACATCAGTAGCGATAGATCTCTGCGGCATGA 97
           |||||..||.|||   ||.|..|||||.|||||.||.||.||.|||||||
           GGCTAGTTTAGTCGTTGAGAGGTCAGTGGCGATTGACCTTTGTGGCATGA 94

           GCCAGGATGAGTTGAATGAGTGCAAACCAGCGGTTAGCAAGGAGAATCCG 147
           ..||||..|||||||||||.|||..|||||||||.||||||.|.||.|||
           CTCAGGCAGAGTTGAATGAATGCTTACCAGCGGTGAGCAAGAATAACCCG 144

           ACGAGCCCATCACAGCCTTGCTGCACCGCTCTGCAACACGCTGATTTGC 197
           |||||||||||....|.|||.||.|.|||||||.||||.|||||.|...|
           ACGAGCCCATCGTTACTTTGTTGTAACGCTCTGAAACATGCTGACTACAC 194

           ATGTCTTTGTGGTTACAAGAACTCTCCATGGCTCGGTTCTTTCGGTGTTG 247
           .|||||||||||.|||||.||||||||.||||||||||||||.|||||.|
           TTGTCTTTGTGGCTACAAAAACTCTCCGTGGCTCGGTTCTTTTGGTGTAG 244

           ATCCTGAACTCGCTTCTGCTCTCCCCAAACAGTGTGGTCTAGCCAACGCC 297
           ||||..|.|||||||||..||||||.|||.||||||..|||.||||||||
           ATCCCAAGCTCGCTTCTAGTCTCCCTAAAGAGTGTGACCTAACCAACGCC 294

           CCAACTTGTTAA 309
           ||||||||||||
           CCAACTTGTTAA 306
```

B

```
DIR1       MASKKAAMVMMAMIVIMAMLVDTSVAIDLCGMSQDELNECKPAVSKENPT 50 At5g48485
           |.|||.| :|:.::|:.:::|:.|||||||||:|.|||||.|||||.|||
DIR1-like  MTSKKVA-IMVIVMVMASLVVERSVAIDLCGMTQAELNECLPAVSKNNPT 49 At5g48490

           SPSQPCCTALQHADFACLCGYKNSPWLGSFGVDPELASALPKQCGLANAP 100
           |||..||.||:|||:.||||||||||||||||||:|||:|||:|.|.|||
           SPSLLCCNALKHADYTCLCGYKNSPWLGSFGVDPKLASSLPKECDLTNAP 99

           TC 102
           ||
           TC 101
```

Supplementary Figure 4.

Alignment of DIR1 (At5g48485) and DIR1-like (At5g48490) sequences.

Complete coding sequences were compared by pairwise alignment using the EMBOSS algorithm. Alignment of DNA sequences is illustrated in **(A)** and alignment of amino acid sequences is shown in **(B)**. Conserved residues are connected by a vertical line (I), similar residues by a double point (:) and divergent residues by a single point (.). Signal peptides are contained within grey boxes and the eight conserved cysteine residues are shown in bold.

A

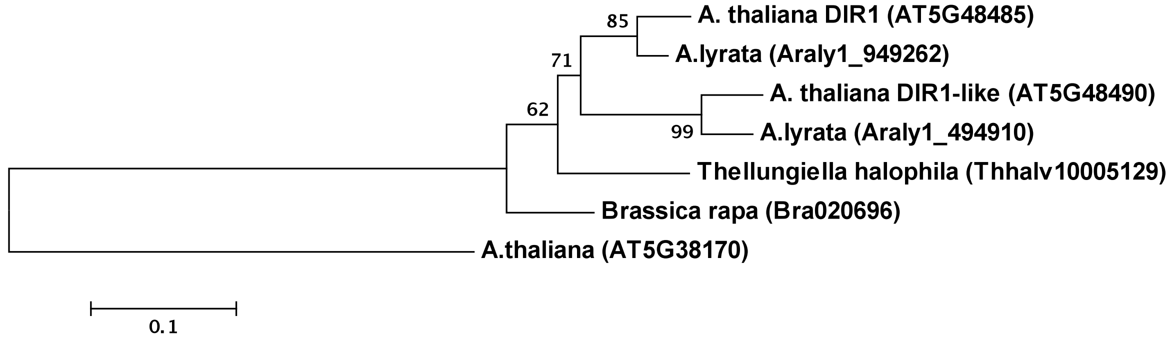

Supplementary Figure 5.
Rooted Phylogenetic Maximum Likelihood tree of DIR1 and DIR1-like proteins.
Protein sequences lacking the divergent ER signal sequence were aligned using Muscle. The evolutionary history was inferred using the Maximum Likelihood method based on the Kimura 2-parameter (Kimura, 1980) model with discrete Gamma distribution using MEGA 5 (Tamura, 2011). 10000 bootstrap replicates were conducted and percent bootstrap values were placed on the branches (Felsenstein, 1985). Branches were drawn to scale, measured in number of substitutions per site and were labeled by species name followed by TAIR gene number or Phytozome 8.0 accession.

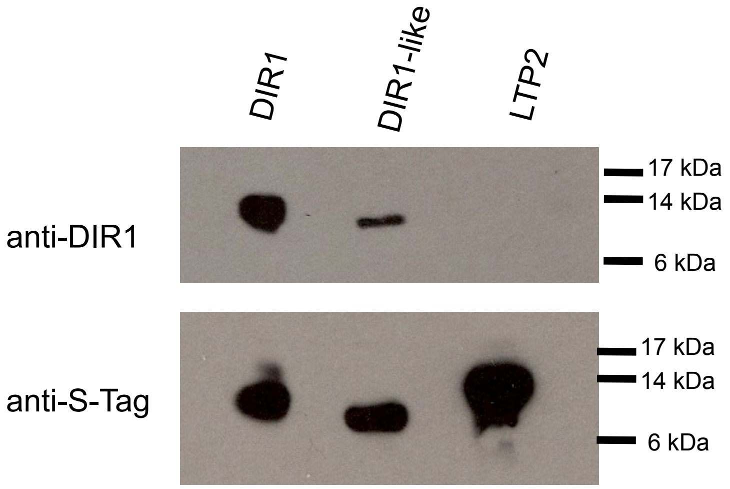

Supplementary Figure 6.
DIR1 antibody specificity. Crude bacterial extracts containing recombinant S-Tag fused DIR1, DIR1-like and LTP2 (AT5G38170) were subjected to protein gel blot analysis and probed with DIR1 antibody and monoclonal S-Tag antibody. Due to different linker sizes between the protein and S-tag, DIR1 is 12.75 kDa while DIR1-like is 9.48 kDa. This experiment was repeated once with similar results observed.

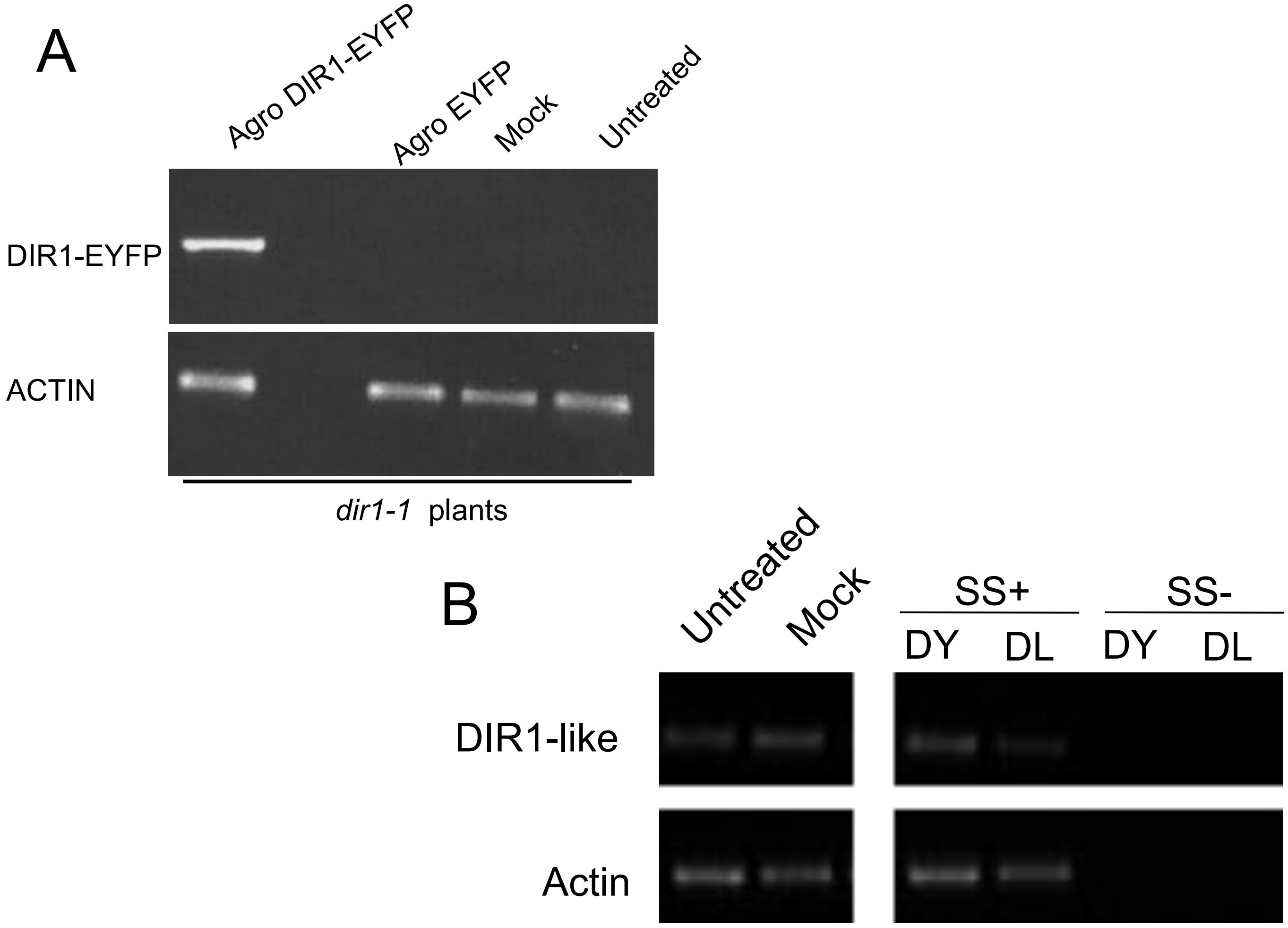

Supplementary Figure 7.

Agrobacterium-mediated transient expression of DIR1-EYFP and DIR1-like

Single leaves were collected 4 days after infiltration with 10^{-6} cfu ml^{-1} *Agrobacterium* harbouring T-DNAs with DIR1-EYFP (DY) or DIR1-like (DL). Untreated leaves and leaves infiltrated with 10mM $MgCl_2$ (Mock) are also shown. See Supplementary Methods for details.

A. RT-PCR analysis of DIR1-EYFP expressed transiently using *Agrobacterium* in *dir1-1* using primers specific to the DIR1-EYFP fusion mRNA.

B. RT-PCR analysis of DIR1-Like Expression in *dir1-1*. Specific DIR1-like primers (same as used in QPCR (See Methods) amplify the endogenous DIR1-like and Agro-expressed DIR1-like. RT controls without SuperScript (SS-) were performed to rule out genomic contamination in intron-less DIR1like. Actin was used as a control RNA levels in each sample.

Primers used:
DYfusion Forward 5'-GGTGTTGATCCTGAACTCGC-3’
DYfusion Reverse 5'-AACTTCAGGGTCAGCTTGCC-3’
DIR1-like Forward 5'-AATGGTGATGGCTAGTTTAGTCGTTGAGAGG-3’
DIR1-like Reverse 5’-TAAACAAACAAAGCAAAACACCATAATGC-3’

Agro-SAR Assay

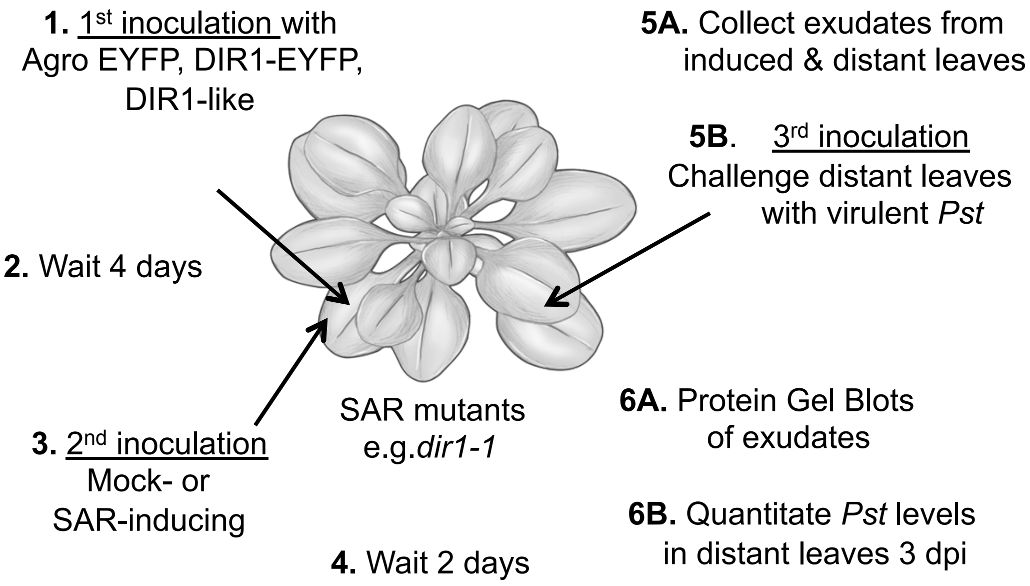

Supplementary Figure 8.

Agro-SAR Assay

1. 1st inoculation of 2 lower leaves with *Agrobacterium* encoding T-DNAs with EYFP, or DIR1-EYP or DIR1-like (0.4 OD_{600}). **2.** The T-DNA with the gene of interest will be transferred and expressed transiently most abundantly by 4 dpi. **3.** 2nd inoculation in the same 2 leaves with 10 mM $MgCl_2$ (mock-inoculation) or SAR-inducing *PstavrRpt2* (10^6 cfu ml^{-1}). **4.** Wait 2 days to induce and establish SAR in distant leaves. **5A.** Petiole exudates can be collected from induced and distant leaves and **5B**. Distant leaves are challenged (3rd inoculation) with virulent *Pst* (10^5 cfu ml^{-1}). **6A**. Perform protein gel blot experiments of petiole exudates and **6B**. Measure the extent of SAR manifestation by quantifying *Pst* levels in distant leaves 3 dpi.

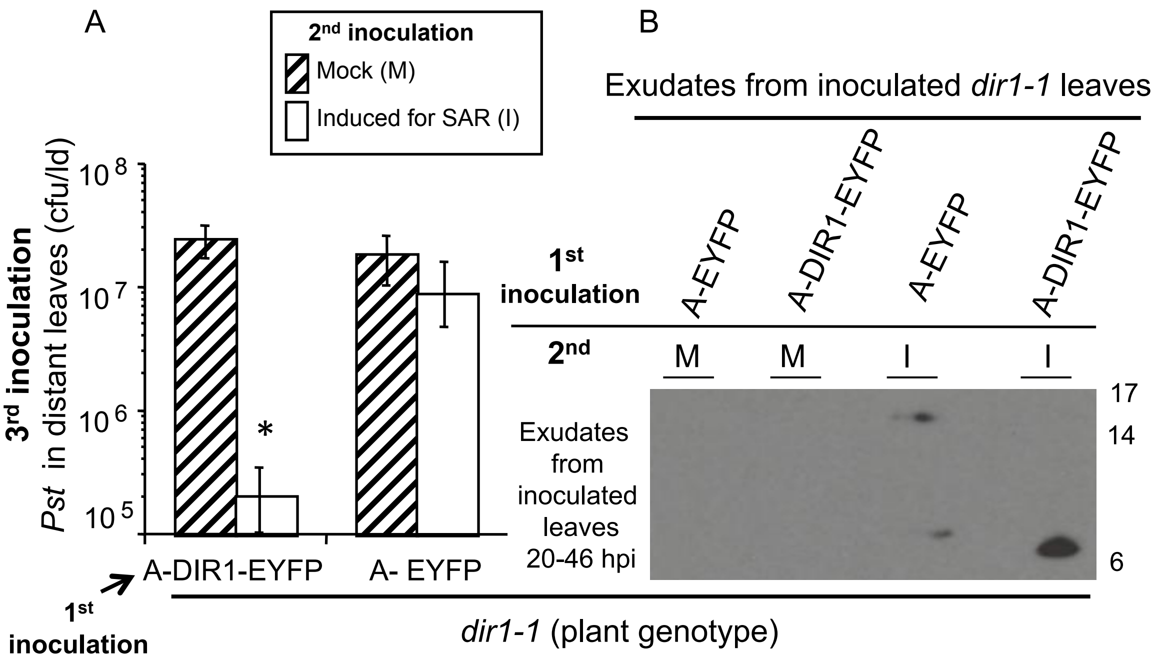

Supplementary Figure 9. The Agro-SAR assay was performed as in Figure 6. Petiole exudates were collected from leaves which received a 1st inoculation with either Agro EYFP or DIR1-EYFP, followed by a 2nd inoculation (Inoc) with either mock (M) or induced for SAR (I) or from distant leaves (Dis) of these same plants. Exudates were lyophilized and subjected to protein gel blot analysis with the DIR1 antibody (B). A & B display a replicate experiment of Figure 6. Protein molecular weight markers are indicated (17,14,6 kDa).

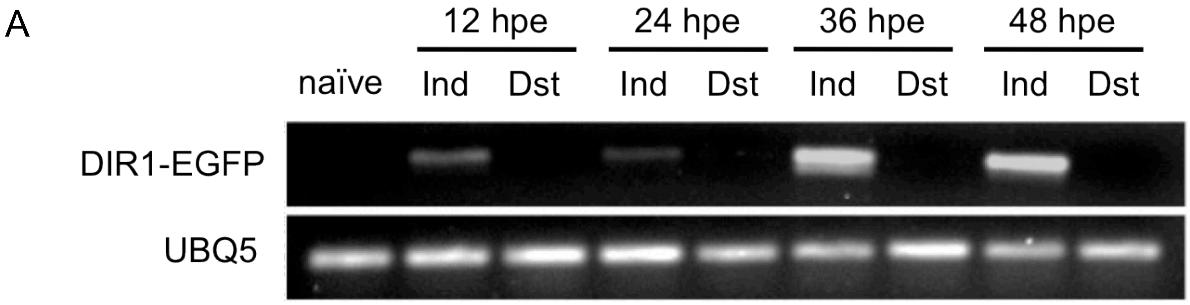

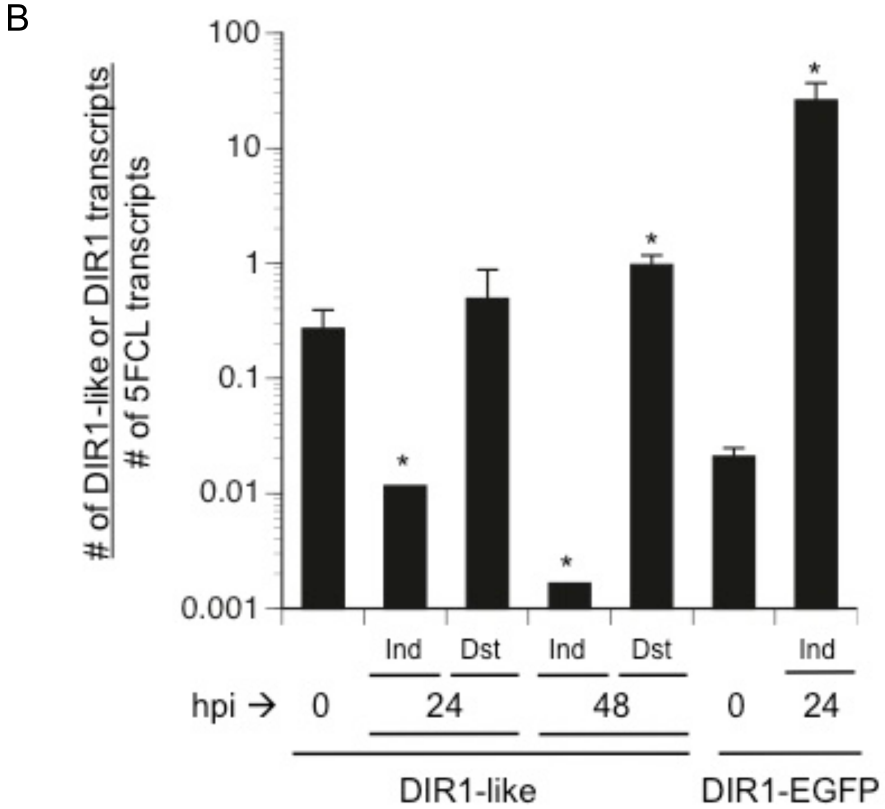

Supplementary Figure 10.

A. RT-PCR analysis of naïve and estrogen treated XVE:DIR1-EGFP/*dir1-1* plants. Expression of the *DIR1-EGFP* fusion was monitored in estrogen-induced (Ind) and distant (Dst) leaves at 12, 24, 36, and 48 hours post estrogen treatment (hpe). *UBQ5* expression was monitored as a control. 26 PCR cycles were used to amplify both *DIR1-EGFP* and *UBQ5*. This experiment has been repeated with similar results.

B. Absolute quantitative Real Time PCR (qRT-PCR) analysis of DIR1-like and DIR1-EGFP in untreated and estrogen-treated XVE:DIR1-EGFP/*dir1-1* lines following SAR induction with *Pst(avrRpt2)* at 0, 24 , 48 hpi in induced (Ind) and distant leaves (Dst). The absolute quantity of transcripts in number of copies per ng of RNA was determined using a standard curve of known template concentrations. The absolute data was normalized to the number of copies per ng of RNA of the reference gene 5FLC. The mean absolute quantity of three biological replicates is presented and error bars represent standard deviation. A Students T-test was used to identify significant differences ($p<0.05$) between 0 and 24 or 48 hpi, denoted by (*). This experiment was repeated with similar results.

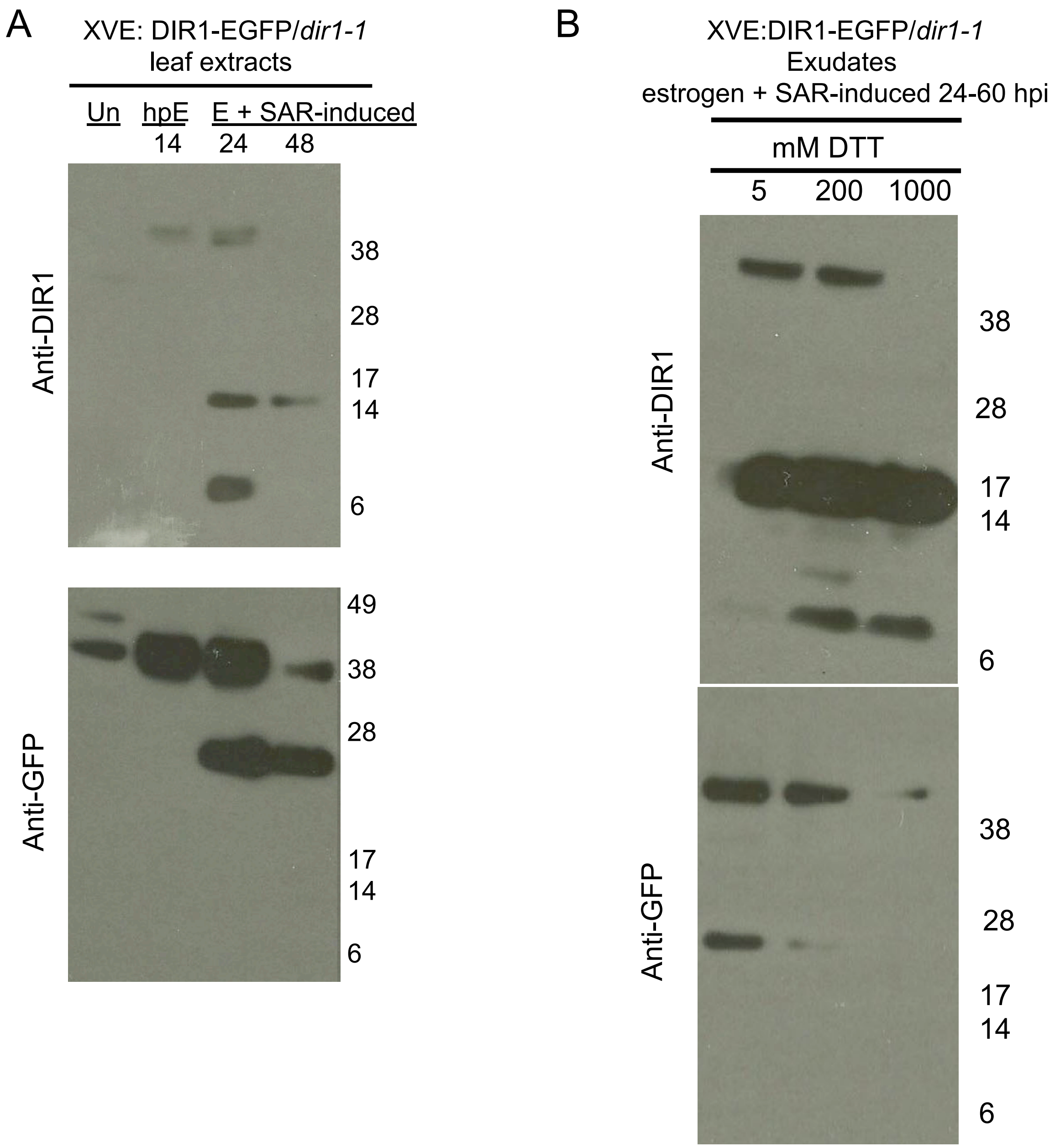

Supplementary Figure 11.
A. Protein gel blots of XVE:DIR1-GFP/*dir1-1* leaf extracts probed with DIR1 and GFP antibodies. Protein molecular weight markers are as indicated. Total protein was extracted from leaves collected from untreated (Un), estrogen-treated at 14 hours post estrogen (hpE), and estrogen + SAR-Induced (with *PstavrRpt2*) plants at 24 and 48 hours post SAR induction.

B. Petiole exudates of estrogen and SAR-Induced leaves collected between 24-60 hours post inoculation (hpi) with *PstavrRpt2*. The reducing-agent dithiothreitol (DTT) was added to the final concentrations indicated above each well. This experiment has been repeated once with similar results.

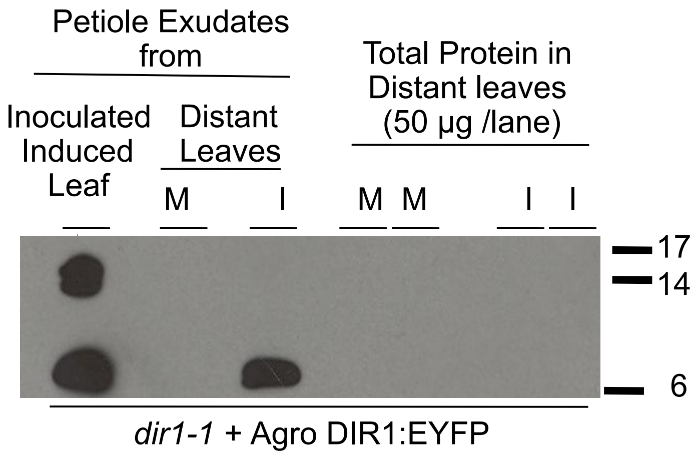

Supplementary Figure 12

Protein gel blot analysis with the DIR antibody of petiole exudates and whole distant leaf extracts from Agro-SAR assays of *dir1-1* plants. See Methods for details.
M- mock-inoculated, I- SAR-induced. Protein molecular weight markers (kDa) are as indicated.

SUPPLEMENTARY METHODS

DIR1-EYFP and DIR1-like *Agrobacterium* Strains

Binary transformation vectors expressing C-terminal EYFP fusion proteins under the control of the 35S promoter were generated using Gateway® technology from Invitrogen. Sequence encompassing full-length *DIR1* but lacking a stop codon was PCR amplified from *Arabidopsis* Ws genomic DNA using forward primer 5’-GGGGACAAGTTTGTACAAAAAAGCAGGCTTAATGGCGAGCAAGAAAGCAGCT and reverse primer 5’-GGGGACCACTTTGTACAAGAAAGCTGGGTTACAAGTTGGGGCGTTGGC *DIR1* lacking its secretion signal sequence but including an engineered start codon was PCR amplified with forward primer 5’-GGGGACAAGTTTGTACAAAAAAGCAGGCTTAATGGCGATAGATCTCTGCGG and the reverse primer described above. EYFP was PCR amplified from pEYFP-N1 (Clontech) with forward primer 5’-GGGGACAAGTTTGTACAAAAAAGCAGGCTTAATGGTGAGCAAGGGCGAGGA and reverse primer 5’-GGGGACCACTTTGTACAAGAAAGCTGGGTTACTTGTACAGCTCGTCCATGCC. PCR products were recombined into entry vector pDONR221 using a BP recombination reaction according to the manufacturer’s instructions. LR recombination reactions were performed according to the manufacturer’s instructions to introduce these coding sequences into plant binary transformation vector p35S-NEYFP, which is based on pMDC83 (Curtis and Grossniklaus, 2003). Resulting plasmids 35S:DIR1-EYFP, 35S: $DIR1_{\Delta}^{1-25}$-EYFP and 35S:EYFP were sequenced, mobilized into *Agrobacterium tumefaciens* strain GV3101/PmP90 by electroporation and transformed bacteria were selected on 2YT medium containing rifampicin, gentamycin and spectinomycin.

Sequence encoding full-length *DIR1-like* was PCR amplified from *Arabidopsis* Ws genomic DNA using forward primer 5’-GGGGACAAGTTTGTACAAAAAAGCAGGCTTAATGACAAGCAAGAAGGTGGC and reverse primer 5’-

GGGGACCACTTTGTACAAGAAAGCTGGGTGTTAACAAGTTGGGGCGTTGG. PCR products were recombined into entry vector pDONR221 and binary destination vector pMDC32 (Curtis and Grossniklaus, 2003) as described above. Plant transformation vector encoding 35S:DIR1-like was mobilized into *Agrobacterium* and selected on solid medium as described above.

XVE:DIR1-EGFP/*dir1-1* and XVE:EGFP/*dir1-1* lines

Estrogen-inducible DIR1-EGFP and EGFP plant expression vectors were constructed using Multisite Gateway technology (Invitrogen 12537-023) in which a 5' element + gene of interest + 3' tag sequences are recombined simultaneously. An estrogen-inducible promoter cassette (XVE) was amplified by PCR using plasmid pER8 (Zuo et al., 2000; acquired from Chua Lab, Rockefeller University) as template with primers F'-GGGGACAACTTTGTATAGAAAAGTTGCTATAGTTTAAACTGAAGGCGGG and R'-GGGGACTGCTTTTTTGTACAAACTTGTGACTAGCTTCAGCGTGTCCTC. The resulting amplicon was recombined into Gateway entry vector pDONRP4-P1R in a BP clonase reaction as described by the manufacturer. EGFP was amplified for use as a carboxy-terminal tag by PCR using plasmid pEGFP-C1 (acquired from Daniel Lab, McMaster University) with primers F'-GGGGACAGCTTTCTTGTACAAAGTGGCTGTGAGCAAGGGCGAGGAGCTG and R'-GGGGACAACTTTGTATAATAAAGTTGTTTATCTAGATCCGGTGGATCC. The resulting amplicon was recombined into entry vector pDONRP2R-P3 in a BP clonase reaction as described. DIR1 sequence lacking a stop codon for translational fusion was amplified by PCR from Ws genomic DNA with primers F'-GGGGACAAGTTTGTACAAAAAAGCAGGCTCTATGGCGAGCAAGAAAGCAGCT and R'-GGGGACCACTTTGTACAAGAAAGCTGGGTCACAAGTTGGGGCGTTGGCTAG. The resulting amplicon was recombined into entry vector pDONR221 in a BP clonase reaction as described. EGFP was also amplified with the addition of three in-frame stop codons by PCR with primers F'-

GGGGACAAGTTTGTACAAAAAAGCAGGCTCTGTGAGCAAGGGCGAGGAGCTG and R'-GGGGACCACTTTGTACAAGAAAGCTGGGTCTTATTATTATCTAGATCCGGTGGATCCCGGG. The resulting amplicon was recombined into entry vector pDONR221 in a BP clonase reaction as described.

XVE:DIR1-EGFP expression vector was completed according to the manufacturer's protocol in a "LR clonase II plus" reaction containing the previously described entry vectors pDONRP4-P1R-XVE, pDONR221-DIR1, pDONRP2R-P3-EGFP and destination vector pH7m34GW (Karimi et al., 2005; acquired from University of Ghent, Belgium). XVE:EGFP expression vector was completed in a LR clonase II plus reaction containing pDONRP4-P1R-XVE, pDONR221-EGFP3stop, pDONRP2R-P3-EGFP and destination vector pH7m34GW. Expression vectors were recovered from *E. coli* and sequenced to verify the integrity of the constructs.

pXVE:DIR1-EGFP and pXVE:EGFP were mobilized into the GV3101 strain of *Agrobacterium tumefaciens* and approximately 20 *dir1-1* plants per construct were subjected to floral dip (Clough and Bent, 1998) for transformation. T1 seeds were sown on MS agar plates supplemented with 20 μg/ml hygromycin, 100 μg/ml cefotaxime and 50 μg/ml β-estradiol (Sigma E8875). Hygromycin resistance was assessed by visual examination of growing seedlings and at 8 days post-germination, seedlings growing on plates were observed under a Leica epifluorescent dissecting microscope.

Hygromycin-resistant seedlings had visible EGFP fluorescence restricted to root tissue, which was an initial indication that β-estradiol was not being transported systemically in plant tissues. 11 XVE:DIR1-EGFP/*dir1-1* and 7 XVE:EGFP/*dir1-1* T1 hygromycin-resistant seedlings were transplanted to soil and T2 seeds were collected from each line. T2 seeds from each line were sown on MS media supplemented as above and screened for segregation of hygromycin resistance and abundant EGFP expression in seedling roots. T3 seeds were collected and similarly screened to verify that EGFP-expressing homozygous lines were established. Two XVE:DIR1-EGFP/*dir1-1* lines (T3-3-5

and T3-2-3), and two XVE:EGFP/*dir1-1* lines (T3-2-5 and T3-9-2) were selected for use in estrogen-SAR assays.

Testing DIR1 antibody specificity by expression in E. coli

DIR1, DIR1-like and LTP2 were cloned into the pET29(b) vector and transformed into Rossetta-Gami 2 *E.coli* (Novagen). Cultures were induced for ectopic expression using 1mM IPTG for 4 hours. Cultures were spun down and resuspended in PBS lysis buffer containing 1 mM PMSF and 1% Triton X-100 followed by sonication for 3x 10 seconds. Lysates were centrifuged and the supernatant representing the soluble crude protein was used in protein gel blot analysis.

References

Clough, S.J. and Bent, A.F. (1998). Floral dip: a simple method for *Agrobacterium*-mediated transformation of *Arabidopsis thaliana*. Plant J. **16**, 735-743.

Curtis M.D. and Grossniklaus, U. (2003). A Gateway cloning vector set for high-throughput functional analysis of genes in planta. Plant Physiol. **133**, 462-469.

Karimi, M., De Meyer, B. and Hilson, P. (2005). Modular cloning in plant cells. Trends Plant Sci. **10**, 103-105.

Kimura M. (1980). A simple method for estimating evolutionary rate of base substitutions through comparative studies of nucleotide sequences. *Journal of Molecular Evolution* **16**:111-120.

Zuo, J., Niu, Q.W. and Chua, N.H. (2000). Technical advance: An estrogen based-receptor transactivator XVE mediates highly inducible gene expression in transgenic plants. Plant J. **24**, 265-273.

Supplementary Table 1

Comparison of SAR Assays –transient expression of EYFP, DIR1-EYFP & DIR1-like (Agro-SAR) or estrogen-induced expression of EGFP or DIR1-EGFP in stable transgenic lines

Expressed Protein	Year	SAR Response[1] / # experiments	SAR^{+} Responses [2] /year or **/all years** (%)	SAR Index[3] /year or **/ave all years**
**Transient-** **Agro-SAR**				
EYFP (endogenous DIR1-like)	2007	0/1	0/1 = 0	0
	2009	0/2 , ++/1	1/3 = 0.3	0.7
	2010	0/4 , ++/1	1/5 = 0.2	0.4
			**2/9 = 0.2 (20)**	**0.4**
DIR1-EYFP	2007[4]	0/2, +/2, ++/1	3/5 = 0.6	0.8
	2008	0/1, +/2, +++/1	3/4 = 0.8	1.3
	2009	+++/3	3/3 = 1.0	3
	2010	0/2, +/1, ++/2	3/5 = 0.6	1
			**12/17 = 0.7 (70)**	**1.5**
DIR1-like	2008	0/1, +/2, ++/1	**3/4 = 0.8 (80)**	**1.0**
**Stable-** **estrogen-induced**				
EGFP (endogenous DIR1-like)	2011	0/2	**0/2 = 0**	**0**
DIR1-EGFP	2011	++++/6	**6/6 = 1**	**4**

[1]SAR Response – statistically significant fold difference (0 = 0-fold, + = 2-fold, ++ = 4-5-fold, +++ = 8-12-fold, ++++ = 20-40-fold) in *Pst* levels in distant leaves of plants that were SAR-induced versus mock-induced.

[2] SAR^{+} Responses include + to ++++

[3] SAR Index = Σ (SAR response x # experiments)/total # experiments for each year or **average for all years**

[4] Example SAR Index Calculation for DIR1-EYFP in 2007- 0/2, +/2, ++/1, SAR Index = (0x2) + (1x2) + (2x1) = 4/5 = 0.8

>ArabidopsisthalianaDIR1(AT5G48485)

------ATAGATCTCTGCGGCATGAGCCAGGATGAGTTGA--------ATGAGTGCAAACCAGCG------GTTAGCAAGGAGAAT-CCGACGAGCCCATCACAGCCT------TGCTGCACCGCTCTGC------
AACACGCTGATTTTGCATGTCTTTGTGGTTACAAGAACTCTCCATGGCTCGGTTCTTTCGGTGTTGATCCTGAACTCGCTTCTGCTCTCCCCAAACAGTGTGGTCTAGCCAACGCCCCAACTTGTTAA

>ArabidopsisthalianaDIR1-like(AT5G48490)

------ATTGACCTTTGTGGCATGACTCAGGCAGAGTTGA--------ATGAATGCTTACCAGCG------GTGAGCAAGAATAAC-CCGACGAGCCCATCGTTACTT------TGTTGTAACGCTCTGA------
AACATGCTGACTACACTTGTCTTTGTGGCTACAAAAACTCTCCGTGGCTCGGTTCTTTTGGTGTAGATCCCAAGCTCGCTTCTAGTCTCCCTAAAGAGTGTGACCTAACCAACGCCCCAACTTGTTAA

>Arabidopsislyrata(XP_002865640)

------ATTGACCTTTGTGGCATGACTCAAGCAGAGTTAA--------ATGAGTGCTTACCAGCG------GTGAGCAAGAATAAC-CCAAAGAGCCCATCACAGCTT------TGTTGCAACGCTCTAA------
AACATGCTGACTACACTTGTCTTTGTGGCTACAAAAACTCTCCGTGGCTCGGTTCTTTCGGTGTAGATCCCAAGCTCGCTTCTGGTCTCCCTAAAGAATGTGACCTAGCCAATGCCCCAGCTTGTTAA

>Arabidopsislyrata(XP_002865639)

------ATTGACCTCTGTGGTATGACACAGGATGAGTTGA--------ATGAGTGCAAACCAGCG------GTTAGCAAGGAGAAT-CCAACAAGCCCATCACAGCCT------TGCTGCTCCGCTCTAC------
AACACGCCGATTTCACCTGTCTTTGTGGCTACAAGAACTCTCCATGGCTCGGTTCTTTCGGTGTTGATCCTGAACTCGCTTCTGGTCTCCCCAAACAGTGTGGCCTAACCAACGCCCCAACTTGTTAA

>Thellungiellahalophila(Thhalv10005129m CDS)

---GCTATTGACCTCTGTGGCATGACCGAGACAGAGTTGA--------ACGAGTGCAAACCAGCG------GTGACCAAGGAGAGT-CCAACGAGCCCATCACAGCTT------TGCTGCGACGCTCTGA------
AACACGCTGACCTCAACTGTCTTTGTGGCTACAAGCACTCTCCATGGCTCGGCTCTTTTGGCATTGACCCCGAGCTAGCTGTTGGACTCCCCACCATATGTGGCCTAGCCAACGCCCCAACTTGTTAA

>**BrassicaRapa(Bra020696)**

---GCGATTGATCTTTGTGGCATGACCCAGTCAGAGTTGA--------ATGAGTGCAAACCAGCG------GTGAGCAAGGAGAAT-CCAACGAACCCATCAACGCTT------TGCTGCGACTATCTGA------
AACACGCTGACATCAGCTGTCTTTGCGGCTACAAGAACTCTCCTTTGCTCGGTTCTTTCGGTATTGATCCGGCGCTCGCTGCTGGACTCCCCACCAAATGTGACATGCCCAACGCTCCAACTTGTTAA

>Arabidopsisthaliana(AT5G38170)

ACTGAGGTCAAACTTTCTGG--------AGGAGAAGCTGATGTAACGTGTGA-
TGCAGTACAGCTTAGTTCATGCGCAACACCAATGCTCACAGGAGTACCACCGTCTACAGAGTGTTGCGGGAAACTGAAGGAGCAACA-GCCG--------
TGTTTTTGTACATATATTAAAGATCCAAGATATAGTCAAT---ATGTTGGTTCTGCAAATGCTAAGAAAACGTTAGCAACTTGTGGTGTTCCTTA---TCCTACTTGTTGA

Supplementary Figure 13. Alignment of Brassicaceae family putative DIR1 othologues.

Using MEGA 5 (Tamura, 2011) a nucleotide Muscle alignment was created of Brassicaceae family members and used to generate the rooted Phylogenetic Maximum Likelihood tree of DIR1 and DIR1-like proteins in supplementary figure 5.
